# What "best practice" could be in Palliative Care: an analysis of statements on practice and ethics expressed by the main Health Organizations

**DOI:** 10.1186/1472-684X-9-1

**Published:** 2010-01-07

**Authors:** Gaia Barazzetti, Claudia Borreani, Guido Miccinesi, Franco Toscani

**Affiliations:** 1 Department of Philosophy University "Vita-Salute San Raffaele" Milan - Italy; 2 Psychology Unit National Cancer Institute Milan - Italy; 3 ISPO Institute for the study and prevention of cancer Florence - Italy; 4 Scientific Director Research Institute for palliative medicine "Lino Maestroni" Cremona - Italy; 5 Current Address: University of Lausanne, EPFL College of Humanities, Switzerland

## Abstract

**Background:**

In palliative care it would be necessary to refer to a model. Nevertheless it seems that there are no official statements which state and describe that model. We carried out an analysis of the statements on practice and ethics of palliative care expressed by the main health organizations to show which dimensions of end-of-life care are taken into consideration.

**Methods:**

The official documents by the most representative health organisations committed to the definition of policies and guidelines for palliative and end-of-life care had been considered. The documents were analysed through a framework of the components of end-of-life care derived from literature, which was composed of 4 main "areas" and of 12 "sub-areas".

**Results:**

Overall, 34 organizations were identified, 7 international organisations, and 27 organisations operating on the national level in four different countries (Australia, Canada, UK and United States). Up to 56 documents were selected and analysed. Most of them (38) are position statements. Relevant quotations from the documents were presented by "areas" and "sub-areas". In general, the "sub-areas" of symptoms control as well as those referring to relational and social issues are more widely covered by the documents than the "sub-areas" related to "preparation" and to "existential condition". Indeed, the consistency of end-of-life choices with the patient's wishes, as well as completion and meaningfulness at the end of life is given only a minor relevance.

**Conclusions:**

An integrated model of the best palliative care practice is generally lacking in the documents. It might be argued that the lack of a fixed and coherent model is due to the relevance of unavoidable context issues in palliative care, such as specific cultural settings, patient-centred variables, and family specificity. The implication is that palliative care staff have continuously to adapt their model of caring to the specific needs and values of each patient, more than applying a fixed, although maybe comprehensive, care model.

## Background

One of the main objectives of a culture is re-orienting death towards life[1]: each person's death threatens society's cohesion by casting a shadow on the feelings of safety and continuity on which every human being bases his/her life and finds support and consolation. For ages the "good death" reflected the community's religious beliefs, and the suffering of the dying person was considered mostly as an unavoidable aspect of the dying process. A good death, in western world, was substantially to die at peace with God and with men[2]. The technological progress of medicine is medicalizing the death more and more, and the gap between the "good" and the actual death has been widening during the last decades[3,4]. Medicine did not deal with dying and death until the birth - some 50 years ago - of the hospice movement, which has the paramount merit of having focused the need of caring for dying persons in order to provide them the best quality of life achievable in their conditions. Actually, the aim of the hospice movement, explicitly or implicitly expressed by its leading persons, is letting terminal patients die better [5,6]. The praxis of palliative medicine, the discipline originated from hospice movement, grounded on scientific approach and rational methods, mainly consists in comprehensive treatments of pain and physical symptoms, in caring for the patient's and their family's needs, and in helping them to face anguish and solitude [7].

Palliative interventions are quite effective on physical suffering. Nevertheless, being free from physical symptoms, even if an important aspect of palliative care practice, is not always enough: psychological, social, emotional and spiritual suffering ought to be also controlled [8]. The non-physical suffering, however, is a much more individual and private matter, and refers to the individual's biography, psychology, beliefs, expectations and cultural mind-set [9,10]. Treating mental and spiritual anguish with the same approach of body problems does not seem that effective and correct, and for some persons, a good death is sometimes missed [11-13].

It seems that an explicit model of best palliative care practice, accepted by all - at least in western Countries - actually does not exist, but the palliative care literature converges towards some specific aspects that contribute to define a death as a good one: symptom control, careful consideration for the social and relational context, preparation to die, and existential wellbeing [14,15]. Thus, care must be centred on the patient's wishes and choices [16-18]: palliative care is, in fact, based on autonomy.

A model of best practice in palliative care should be flexible and discussable, and, specially, manifold. It is hardly maintainable that a unique model can be used in a world of moral and cultural strangers [19-21] given that what makes death "good" is different for everyone. In reality, inside hospice movement and palliative care, there are no official statements which state and describe that model. It is, however, probable that the practice of palliative care is actually grounded on a implicit model.

Recently, the category of "good death" as an outcome of palliative medicine has been broadly discussed [7,22-31]. Furthermore the hospice movement has a strong stand against euthanasia and assisted suicide [32-34]. The querelle between palliativists and the right-to-die movement is actually on the limits that ought to be set on the personal autonomy, notwithstanding the common cultural roots of both parties -i.e. "the right of individuals to make their own choices about how they should live and die" [9].

In order to understand if an implicit model of best practice in palliative care does exist, we carried out a qualitative analysis of the statements on practice and ethics of palliative care expressed by the main health organizations to show which dimensions of end-of-life care are taken into consideration.

## Methods

This qualitative study aims at investigating the notion of "best palliative care practice" arising from the official documents by the most representative health organizations committed to the definition of policies and guidelines for palliative and end-of-life care.

The organizations and their documents were selected on the basis of the following three criteria:

- The organization is representative (e.g. on an international or on a national level) of several associations or of professional groups involved in health care.

- The organization has produced documents on ethical, physical and psycho-social issues related to end-of-life care.

- The documents analysed focus on the general practice of palliative care, pain relief and the care of dying patients in general, or deal with more specific end-of-life issues, such as euthanasia, assistance of patients in a permanent vegetative state, sedation at the end of life, and the use of nutrition and hydration, assisted suicide.

The selection and analysis of the documents have been carried out in two phases: a first survey was completed in 2007; this first survey was updated in 2008 in order to find out recently published documents, as well as revisions of the documents included in the first survey.

The procedure adopted for finding the documents combined two methods:

- A retrieval of the directories of organizations available on the websites of the

International Association for Hospice and Palliative Care (IAHPC Directory: http://www.hospicecare.com/yp/) and of the European Association for Palliative

Care (EAPC Directory: http://www.eapcnet.org/organisations/OffBodies&Ass.html), which allowed to identify several organizations that produced documents.

- A document research on the web (Google search: "position statement" AND ("dying" OR "end of life care" OR "good death" OR "palliative care")), by which it was possible to find out additional documents from a number of organizations that were not included in the directories of the IAHPC and of the EAPC.

The documents were classified as:

a) Documents of palliative care institutions, or other medical or health institutions;

b) Documents on end-of-life/terminality in general, or on a specific situation/need/symptom of end-of-life;

c) Documents classifying themselves as "position statement" (in the title), or "others".

The documents were analysed through a framework of the components of end-of-life care which was developed on the basis of a literature search in a previous work [33]. That review included both qualitative studies on the perceptions of a "good death" amongst patients, caring staff and family members, and more theoretical studies on the origins and the history of the notion of "good death". This thematic grid, which has been updated with the most recent citations [11,14,19,30,35-64], was composed of 4 main "areas" (i.e. A, B, C and D) and of 12 "sub-areas" (i.e. A1, A2, etc.) (see table 1).

**Table 1:** Thematic grid

**A**	**SYMPTOMS**
A1	Pain and symptom control
A2	Control of anxiety and other psychological symptoms (not dying with fear)
A3	Being assisted by a staff in order to make the process of dying more comfortable
**B**	**RELATIONAL AND SOCIAL AREA**
B1	Respect of cultural values and individual preferences
B2	Emotional support provided to the family
B3	Good communication among patient/families/close friends/caring staff
B4	Having close people nearby/family acceptance of the patient's condition/not feeling a burden for family and friends
**C**	**PREPARATION**
C1	Importance given to preparation/awareness of diagnosis/awareness of dying
C2	Choice of place of dying
C3	Maintaining a sense of control (the possibility of controlling relevant aspects of one's own existence and/or deciding what and when to delegate to others); maintaining a dimension of continuity of life right to the end
**D**	**EXISTENTIAL CONDITION**
D1	Being at peace with oneself/finding meaning
D2	Spiritual needs/Religious practices

The content analysis of the documents was carried out with a view to finding out in the texts the various areas and sub-areas of the framework.

## Results

Overall, 34 organizations were identified, i.e. 7 international organizations, and 27 organizations operating on the national level in four different countries (Australia, Canada, UK and United States). Fifty-six documents were selected and analysed.

Additional file 1 provides a list of the documents, including the reference to the name and the level of representativeness (international, or national) of the organization which produced the document, and the code assigned to the document in the course of texts analysis. Moreover, the table indicates the various types of documents selected for this study: most of them (38) are position statements.

Additional file 2 shows all the relevant quotations from the documents analysed. The attachment consists of several tables sorting quotations by "areas" and "sub-areas", in order to illustrate the specific quotes referring to the elements of the framework.

The presence and the specific meaning of sub-areas in the documents are reported in the following.

### A - SYMPTOMS

#### A1 - Symptom control

There is a large convergence of most documents on symptom control, in particular on pain. Pain treatment is as important for doctors and for nurses. The importance of an impeccable early symptom assessment before treating is highlighted. There is a different emphasis on the expected results of symptom control in the documents: some of them refer to "freedom" from pain (e.g. WHO I, EAPC II), whereas others are focussed on "managing" (e.g. WHO II, USA ACS, USA AMA) or "alleviating", "easing" and "mitigating" them (e.g. ICN, ESMO, CANADA CHPCA I, USA NHPCO I). Physical pain is mostly considered as a part of a broader condition of suffering, thus accepting the concept of "total pain" as the specific connotation of the terminal patient.

#### A2 - Control of anxiety and other psychological symptoms (not dying with fear)

Psychological suffering is part of the "total pain" and is, as well as the physical symptoms, an objective of the palliative caring. Some of the documents refer specifically to anxiety and depression (i.e. WHO II, CANADA CHPCA I, USA AGS, USA AMA, USA ASCO I, AUSTRALIA ANZSPM I); others describe it as a broader constellation of discomforts associated with impending death (e.g. WMA I, USA AAHPM IV, USA ONS II). These are frequently linked to spiritual or social problems. Some documents, interestingly, consider the psycho-social dimension as a need (e.g. WHO IV, USA ONS II). This perspective implies the search for strategies of need-satisfaction rather than of symptom "sedation".

#### A3 - Being assisted by a staff in order to make the process of dying more comfortable (both physical and psychological)

In general, accepting the palliative care goal of making the dying process as easy as possible, the documents highlight the role of a multidisciplinary team, with special knowledge and skills, in order to deal with the problems and needs of the patients and of their families.

### B - RELATIONAL AND SOCIAL AREA

#### B1 - Respect of cultural values and individual preferences

Among the most important elements of caring are the acknowledgement of personal, social, religious and cultural values and beliefs, of both patients and families, as well as the patients' choices about the end-of-life caring. This implies paying special attention to their identification, and respecting and not judging them. One of the documents (i.e. AUSTRALIA PCA II) suggests that also deliberate requests of ending life have to be respected, should they reflect the patient's wishes. Another document (i.e. USA AAFP I) advocates for the availability of instruments that might permit the empowerment of the patients and the respect of their choices.

#### B2 - Emotional support provided to the family

This is a common topic in the relational and social area. Family, in fact, is an object of care, together with the patient. Family must be supported also after patient's death. Some documents (e.g. USA AAP, UK NCPC, UK SC, AUSTRALIA AMA) emphasize the importance of a support that should include specific measures such as counselling, in order to help the family to successfully cope with the patient's illness

#### B3 - Good communication between patient/families/close friends/caring staff

Communication is a crucial element of care. It must be open, honest, understandable, and must be given in an atmosphere of sensitivity and compassion with adequate emotional support. At the end-of-life, communication concerns the symptoms, their cause and treatment options, as well as issues related to death and dying. Some documents (e.g. USA ASCO II) claim for a health professionals' specific training. One of the documents points out that nurses should advocate for the communication of the patient's preferences across the various health-care settings (i.e. CANADA CNA).

#### B4 - Having close people nearby/Family acceptance of the patient's condition/Not feeling a burden for family and friends

Family is acknowledged as a crucial element of end-of-life care, but this care must not become an unbearable burden. The care must be freely and consciously accepted and carried out by the relatives. The appropriate climate for a dying person ought comprehend the following elements: physical and emotional closeness; acceptance of death; providing the patient does not feel her/himself as a burden for the caregivers.

### C - PREPARATION

#### C1 - Importance of preparation/awareness of diagnosis/awareness of prognosis (awareness of dying)

The analysis of the documents has shown that the awareness of diagnosis and prognosis (and of impending death) is not considered as relevant. Amongst the international organizations, only one (i.e. WHO I) acknowledges the importance of preparation. In the documents that take this into account, the term "preparation" does not exclusively refer to death, but more often to the dying process. In general, these documents recommend paying a thoughtful attention to the patient's verbal and non-verbal communication in order to understand when and if that very patient is ready to deal with these subjects; and to let the patient feel that the caregiver too is ready to give her/him every explanation and answer.

#### C2 - Choice of place of dying

Among the few documents that consider this issue, five (i.e. WHO IV, CANADA CHPCA I and II, USA AAHPM IV, and USA AGS) refer to the setting of care in the last phases of life, and four documents (i.e. CANADA CNA, USA AAP, USA AMA, AUSTRALIA CARNA) refer to the place of death. No specific setting is considered as the most suitable a priori, whether it is the place where the final days of life have to be spent, or the place where death will occur: the place ought be chosen on the patient's preference and/or needs.

#### C3 - Maintaining a sense of control (possibility of controlling relevant aspects of one's own existence and/or deciding what and when to delegate to others)/Keeping a dimension of continuity of life right to the end

The relevance given to the patient's empowerment is very high. It is important that the patient is helped to keep the control on the dying process by means of: an adequate and effective support; the share of the decision-making; the exploitation of her/his resources; the respect of her/his freedom of choice; advanced directives.

### D - Existential condition

#### D1 - Being at peace with oneself/finding meanings

Only a few documents take this issue into account. For those nearing the end of life, impending death could be an opportunity to give meaning to the disease and/or to their life. Thus, the caregivers have to help the patient to this task.

#### D2 - Religious or spiritual practices

The assessment of spiritual and religious needs is considered as a relevant element of a good end-of-life care. The caregivers are committed to acknowledge the spiritual needs and to facilitate the accomplishment of specific religious practices. One of the documents (i.e. USA HPNA III), focusing on spiritual care at the end of life, emphasizes the importance of acknowledging and supporting patient's spiritual beliefs and expressions, and recognizes the patient's right to decline religious support.

The analysis of the documents led to the identification of additional key-elements of end-of-life care that were not included in the framework taken from the review of literature.

A description of the additional areas and sub-areas arising from the statements is provided in the following.

### E - End-of-life decisions

#### E1 - Death as natural or normal/Not to hasten nor to postpone death

Overall, 11 documents consider death as a "normal" process and "a natural part of life progression", and 9 documents explicitly refuse any kind of medical intervention directed to "hasten" or to "postpone" death. In 5 documents the rationale behind the refusal of interventions that deliberately hasten death is that dying has to be considered as a normal part of the life process. There is a nuanced difference between the documents stating that palliative care should not accelerate nor delay death (e.g. WHO I, EAPC I, UK SC), and documents affirming that palliative care does not intend to accelerate nor postpone death (e.g. USA HPNA I, USA ONS I). Interestingly, one document contends that the naturalness of death is compatible with declining or withdrawing futile treatments (i.e. AUSTRALIA PCA II). Two of the documents refer to the rule of "double effect" to justify the use of pain medication which might have a secondary and unintended effect of hastening death (i.e. CANADA CNA, USA HPNA I).

#### E2 - Death as an unwanted effect of sedation/Withdrawing or withholding treatments/Euthanasia and assisted suicide

Euthanasia and assisted suicide are considered unethical, but terminal pharmacological deep sedation and the (even if rare) life shortening due to effective/high doses of analgesics and/or sedatives are not to be considered as euthanasia (e.g. USA AAHPM V, USA HPNA I). In general, the withdrawing or the withholding of treatments are considered as acceptable measures only if treatments do not effect any amelioration of the patient's condition, but merely prolong the process of dying (e.g. WHO I, EAPC II, USA AMA). One of the documents (e.g. USA ACS) highlights the doctor's responsibility of sparing futile treatments in every situation that involves imminent dying. Nevertheless, several documents clearly state that withdrawing and withholding treatments (including life-sustaining measures) should be consistent with the patient's wishes (e.g. WMA III, CANADA CHPCA II, USA AAHPM II). Amongst these documents, one clearly states that artificial nutrition and hydration should be considered as any other treatments and might be withhold or withdrawn when doing so is consistent with the patient's preferences (i.e. USA NHPCO IV).

#### E3 - Participation in the decision-making process

All documents maintain that patients should be involved in every decision concerning treatments. Up to 12 documents by international and national organizations (e.g. WMA I, USA HPNA I, USA NHPCO IV, CANADA CHPCA I, AUSTRALIA AMA) clearly state that patients have a "right" to make informed decisions on treatments, including the right to refuse treatments. Five of the documents (i.e. CANADA CHPCA II, CANADA CNA, USA AAHPM II, USA AMA, USA NHPCO IV) provide indications on advanced directives, formal living will, the designation of proxy decision-makers, which are considered as a means to collect and honour the patient's choices. Interestingly, one of the documents (e.g. EAPC II) supports a more nuanced participation of the patient in the decision-making process, thus referring to a specific time in the disease progression, when it is right to honour the patient's refusal of treatment that prolong suffering without any gain for the patient's condition.

### F - Quality of life

A considerable number of documents consider quality of life as the main goal of care at the end of life. This goal is so important that is licit to forgo any other result, including prolonging life or keeping the patient alive. Most of these documents assume that quality of life is a relevant parameter of an effective palliative care. In particular, three of the documents maintain that quality of life should be defined by each patient (i.e. CANADA CHPCA II) and by his/her family (i.e. CANADA CHPCA I, AUSTRALIA CARNA). Only one document (i.e. WHO I) acknowledges the need of instruments to measure the quality of remaining life, and provides a list of items that should be evaluated in order to establish it. All the other documents do not provide a description of how to assess the quality of life of patients facing impending death. Some documents (i.e. WHO I, WHO V, CANADA CHPCA I, UK NCPC) explicitly refer to the quality of life of family members taking care of patients who suffer a life-threatening illness. One of the documents details specific therapies that might improve the patient's quality of life (i.e. USA AAP).

### G - Dignity

A few documents refer to the issue of dignity, although the meaning of this term is altogether nuanced and variable. Some documents (i.e. ICN, CANADA CHPCA I, USA ANA) refer to a "dignified death", while others allude to a general "sense of dignity" (i.e. CANADA CHPCA II) or to the possibility of maintaining "dignity and independence" (i.e. USA AGS) as something that should be guaranteed to dying patients. One of the documents affirms that the caring staff should approach death in a way that it "dignifies life" (i.e. UK SC). In general, a specific definition of the term "dignity" is lacking.

## Discussion

Analysis of the documents shows that all the dimensions of end-of-life care found in the literature and included in the framework (see Table 1: Thematic grid) are echoed in the statements of the most representative organizations committed to the definition of policies and guidelines for palliative and end-of-life care. It is worth noting that all the national organizations found according to our research strategy belong to English speaking countries. This might be due to the fact that it was in these countries that the palliative care movement first developed and flourished in the 60s and 70s.

In general, the "sub-areas" of symptom control (i.e. A1, A2 and A3) as well as those referring to relational and social issues (i.e. B1, B2, B3 and B4) are more widely covered by the documents than the "sub-areas" related to "preparation" (i.e. C1, C2 and C3) and to "existential condition" (i.e. D1 and D2). This result is consistent with what is stated by several studies showing that the control of symptoms and of the psychosocial dimension of dying [15,17,25,26,35-41], is given a higher relevance than the control of the dying process by the patient himself [15,19,25,26,34,51,55].

With regard to symptoms, the control of pain and of psychological distress (i.e. A1 and A2) is acknowledged as fundamental, while being assisted by a staff member in order to make the process of dying more comfortable (i.e. A3) is considered as less relevant. This result seems to be counteracted by the evidence from the literature, which shows that being comfortable is seen as important both by patients and by health care professionals [59].

As to the relational and social dimension, a large number of documents state that individual preferences as well as personal values and beliefs (i.e. B1) should be respected and honoured. This issue has been extensively discussed in the literature [4,12,23,25,51,56,60] and is particularly relevant for patients sharing cultural values which are different from those dominant in society [17].

Most documents combine the respect for personal beliefs and values with the importance of addressing one's spiritual needs and of facilitating religious practices (i.e. D2), thus showing consideration for individual preferences both from the relational and from the existential perspective. However, the importance attributed to respect for individual preferences seems to be in contradiction with the minor weight lent to the choice of the place of dying (i.e. C2).

Further discrepancies can be found between issues related to preparation and issues related to the relational and social dimension of dying. Indeed, many documents recognise the importance of good communication between the patient and the caring staff (i.e. B3), and state that communication should include information about diagnosis and prognosis, as well as the discussion of issues related to death and dying. Yet, this result jars with the fact that only a few documents refer to the awareness of diagnosis and of impending death (i.e. C1), an omission which is even more striking since how often Western surveys address this issue [4,12,23,25,51,53,57,60].

It might be argued that, due to the discrepancies between the element of preparation and the relational and social area, it is not possible to derive from the documents an integrated model of best palliative care practice. In particular, it might be suggested that the documents do not offer a coherent model for policies directed to the actual empowerment of patients in the decision-making process. This is especially evident with regard to end-of-life decisions. While there is a general agreement between the documents that patients should take part in end-of-life choices, withdrawing and withholding of life-sustaining treatments are viewed as the result of an evaluation that is mainly up to the doctor. Indeed, the consistency of end-of-life choices with the patient's wishes is given only a minor relevance.

Another result that is worth discussing is the small relevance given to being in control of oneself (i.e. C3). Only one third of the documents refers to this item, whereas it is one of the most important elements of end-of-life care present in the literature [59]. This might be due to the fact that the maintenance of control is a multifaceted and a patient centred issue, which can only with difficulty be addressed by policy statements on palliative and end-of-life care. The same conclusion may be suggested for completion and meaningfulness at the end of life (i.e. D1), which is given a minor relevance in the documents, while several qualitative studies on patients, families and caregivers account for this item [12,17,23,56,60,62].

On the other hand, there seems to be no persuasive explanation to the fact that minimising the burden of caring on the family is hardly covered in the statements. While, according to the literature, freedom from financial and physical burden is considered by patients and caregivers as one of the most important component of a end-of-life care [17,56,58,59,61] only a few documents address this issue. It might be suggested that, in general, the statements tend to consider "financial and physical" support to the family as less relevant than "emotional" support. In fact, a great number of documents consider emotional support to the family (i.e. B2) as reasonably important. Nevertheless, this consideration does not prevent from observing that minimizing the burden of care is generally underestimated in the documents.

Finally, it is worth noting that a definition of quality of life at the end of life is lacking, whereas this issue is widely covered by the documents. Of course, the concept of quality of life is highly individual and fluid, and it might be difficult to give a precise definition of this notion. However, several studies have already proved the possibility of developing instruments to assess both the quality of life for dying patients and the quality of care at the end of life [52,57,59,63,64]. Therefore, it might be observed that the documents generally fail to address the issue of quality of life in a consistent and precise manner.

## Conclusions

This work demonstrates that all the dimensions of end-of-life care stemming from the literature are reflected in the official documents by the most representative organizations committed to the definition of guidelines for the care of the dying patients. A few additional items emerged from the analysis of the documents, thus completing the framework which was formerly taken from the literature. The resulting grid (see Table 2: New thematic grid) consists of a more comprehensive framework, which might facilitate the assessment of the high quality palliative care process.

**Table 2:** New thematic grid

**A**	**SYMPTOMS**
A1	Pain and symptom control
A2	Control of anxiety and other psychological symptoms (not dying with fear)
A3	Being assisted by a staff in order to make the process of dying more comfortable
**B**	**RELATIONAL AND SOCIAL AREA**
B1	Respect of cultural values and individual preferences
B2	Emotional support provided to the family
B3	Good communication among patient/families/close friends/caring staff
B4	Having close people nearby/family acceptance of the patient's condition/not feeling a burden for family and friends
**C**	**PREPARATION**
C1	Importance given to preparation/awareness of diagnosis/awareness of dying
C2	Choice of place of dying
C3	Maintaining a sense of control (the possibility of controlling relevant aspects of one's own existence and/or deciding what and when to delegate to others); maintaining a dimension of continuity of life right to the end
**D**	**EXISTENTIAL CONDITION**
D1	Being at peace with oneself/finding meaning
D2	Spiritual needs/Religious practices
**E**	**END-OF-LIFE DECISIONS**
E1	Death as natural or normal/Not to hasten nor to postpone death
E2	Death as an unwanted effect of sedation/Withdrawing or withholding treatments/Euthanasia and assisted suicide
E3	Participation in the decision-making process
**F**	**QUALITY OF LIFE**
**G**	**DIGNITY**

On the other hand, an integrated model of best palliative care practice is generally lacking in the documents. It might be argued that the lack of a fixed and coherent model is due to the relevance of unavoidable context issues in palliative care, such as specific cultural settings, patient-centred variables, and family specificity. The implication is that palliative care staff have continuously to adapt their model of caring to the specific needs and values of each patient, more than applying a fixed, although maybe comprehensive, care model.

## Competing interests

The authors declare that they have no competing interests.

## Authors' contributions

GB performed the search and analysis of the documents. CB and GM participated in the analysis and wrote the different versions of the paper. FT discussed the results and wrote the background section. All authors read and approved the final manuscript.

## Pre-publication history

The pre-publication history for this paper can be accessed here:

http://www.biomedcentral.com/1472-684X/9/1/prepub

## Supplementary Material

Additional file 1 **list of documents**. list of documents with name and level of representativeness of the organizations, and code assigned for the text analysis. Click here for file

Additional file 2 **quotations**. quotations from selected documents sorted by areas and subareas. Click here for file
