# Supplementary material for: What "best practice" could be in Palliative Care: an analysis of statements on practice and ethics expressed by the main Health Organizations
Source: BMC Palliat Care. 2010 Jan 7;9:1. doi: 10.1186/1472-684X-9-1 (PMC2823604; doi:10.1186/1472-684X-9-1)
Supplement: Additional file 1 — list of documents. list of documents with name and level of representativeness of the organizations, and code assigned for the text analysis. [file 1472-684X-9-1-S1.DOC]

**Attachment 1: Table of documents**

The table provides a list of the documents, including the reference to the name and the level of representativeness (international or national) of the organisation that has produced the document, and the code assigned to the document during the process of analysis of text. Moreover, the table indicates the various types of documents selected for this study.

Legenda of “Further classification”:

Type of institution: Palliative Care institution: PCI; Other health/medical institution: HI

Subject: End-of-life/terminality in general: EOL; Specific situation/need/symptom of end-of-life: SPEC

Type of document: Position Statement: PS; Other: O.

| **Representativeness**  **of the organization** | **Name of the organization** | **Title of the document**  **and date of delivery** | **Further Classification** | **Document**  **code** |
| --- | --- | --- | --- | --- |
| International | World Health Organization | Cancer pain relief and palliative care (1990) | HI; EOL; O | WHO I |
|  |  | Cancer pain relief (1996^2^) | HI; SPEC; O | WHO II |
|  |  | Symptom relief in terminal illness (1998) | HI; SPEC; O | WHO III |
|  |  | Cancer pain relief and palliative care in children (1998) | HI; EOL; O | WHO IV |
|  |  | National cancer control programs: policies and managerial guidelines (2002^2^) | HI; EOL; O | WHO V |
|  | World Medical Association | World Medical Association Declaration on Terminal Illness (2006) | HI; EOL; O | WMA I |
|  |  | World Medical Association Declaration on Euthanasia (1987) | HI; SPEC; O | WMA II |
|  |  | World Medical Association Statement on Physician-Assisted Suicide (1992) | HI; SPEC; PS | WMA III |

| **Representativeness**  **of the organization** | **Name of the organization** | **Title of the document**  **and date of delivery** | **Type of the document** | **Document**  **code** |
| --- | --- | --- | --- | --- |
| International | Academy of Psychosomatic Medicine | Psychiatric Aspects of Excellent End-of-Life Care (2005) | HI; EOL; PS | APM |
|  | European Association for Palliative Care | Definition of palliative care (1989, revised in 1998) | PCI; EOL; O | EAPC I |
|  |  | Roy DJ, Rapin C-H, the EAPC Board of Directors, Regarding euthanasia, *European Journal of Palliative Care* 1994; 1: 57-59. | PCI; SPEC; O | EAPC II |
|  | European Society for Medical Oncology | ESMO Policy on Supportive and Palliative Care | HI; EOL; PS | ESMO |
|  | International Council of Nurses | Nurses’ Role in Providing Care to Dying Patients and their Families (2000, 2006) | HI; EOL; PS | ICN |
|  | International Society of Psychiatric Nurses | ISPN Position Statement on Palliative Care | HI; EOL; PS | ISPN |

| **Representativeness**  **of the organization** | **Name of the organization** | **Title of the document**  **and date of delivery** | **Type of the document** | **Document**  **code** |
| --- | --- | --- | --- | --- |
| National  (CANADA) | Canadian Hospice Palliative Care Association | Hospice Palliative Care Nursing Standards of Practice (CHPCA Nursing Standards Committee 2002) | PCI; EOL; O | CANADA  CHPCA I |
|  |  | A Model to Guide Hospice Palliative Care (2002) | PCI; EOL; O | CANADA  CHPCA II |
|  | Canadian Nurses Association | Position Statement. Providing Nursing Care at the End of Life (2008) | HI; EOL; PS | CANADA CNA |
|  | Canadian Pain Society | Position Statement on Pain Relief (1997) | HI; SPEC; PS | CANADA CPS |
| National  (USA) | American Academy  of Family Physicians | Core Principles for End-of-Life Care (2000, 2006) | HI; EOL; PS | USA AAFP I |
|  |  | End-of-Life Care (1997, 2006) | HI; EOL; PS | USA AAFP II |
|  | American Academy of Hospice and Palliative Medicine | Definitions of Palliative Care and Palliative Medicine | PCI; EOL; O | USA AAHPM I |
|  | American Academy of Hospice and Palliative medicine | Statement on Palliative Sedation (2006) | PCI; SPEC; PS | USA AAHPM II |
|  |  | Statement on Artificial Nutrition and Hydration Near the End of Life (2006) | PCI; SPEC; PS | USA AAHPM III |
|  |  | Statement on Comprehensive End-of-Life Care and Physician-Assisted Suicide (1997) | PCI; EOL; PS | USA AAHPM IV |
|  |  | Statement on Clinical Practice Guidelines for Quality Palliative Care (2006) | PCI; EOL; PS | USA AAHPM V |
|  | American Academy of Pediatrics | Palliative Care for Children (2000) | HI; EOL; PS | USA AAP |
|  | American College  of Surgeons | Statement on principles guiding care at the end of life (1998) | HI; EOL; PS | USA ACS |

| **Representativeness**  **of the organization** | **Name of the organization** | **Title of the document**  **and date of delivery** | **Type of the document** | **Document**  **code** |
| --- | --- | --- | --- | --- |
| National  (USA) | American Geriatrics Society | The care of the dying patients (AGS Ethics Committee: 2002, 2007) | HI; EOL; PS | USA AGS |
|  | American Medical Association | Statement on end-of-life care (2005) | HI; EOL; PS | usa ama |
|  | American Nurses Association | Pain Management and Control of Distressing Symptoms in Dying Patients (2003) | HI; SPEC; PS | USA ANA |
|  | American PainSociety | Treatment of Pain at the End of Life: A Position Statement from the American Pain Society (1997, 2006) | HI; SPEC; PS | USA APS |
|  | American Society for Pain Management Nursing | Position Statement on Pain Management at the End of Life (2003) | HI; SPEC; PS | USA ASPMN |
|  | American Society of Clinical Oncology | Cancer Care During the Last Phase of Life, *Journal of Clinical Oncology* 1998; 16: 1986-1996. | HI; EOL; O | usa  asco i |
|  |  | End of Life Care (1998) | HI; EOL; PS | usa asco II |
|  | Hospice & Palliative Nurses Association | HPNA Position Statement Palliative Sedation (2003) | PCI; SPEC; PS | USA HPNA I |
|  |  | HPNA Position Statement Pain (2003) | PCI; SPEC; PS | USA HPNA II |
|  |  | HPNA Position Statement Spiritual Care (2006) | PCI; SPEC; PS | USA HPNA III |

| **Representativeness**  **of the organization** | **Name of the organization** | **Title of the document**  **and date of delivery** | **Type of the document** | **Document**  **code** |
| --- | --- | --- | --- | --- |
| National  (USA) | National Consensus Project for Quality Palliative Care | Clinical Practice Guidelines for Quality Palliative Care | PCI; EOL; O | USA NCP |
|  | National Hospice and Palliative Care Organization | Hospice Philosophy Statement (2000) | PCI; EOL; PS | USA NHPCO II |
|  |  | Preamble to NHPCO Standards of Practice (2000) | PCI; EOL; PS | USA NHPCO I |
|  |  | Hospice and Palliative Care Code of Ethics (2002) | PCI; EOL; O | USA NHPCO III |
|  |  | Commentary and Position Statement on Artificial Nutrition and Hydration (2005) | PCI; SPEC; PS | USA NHPCO IV |
|  |  | Commentary and Resolution on Physician Assisted Suicide (2005) | PCI; SPEC; PS | USA NHPCO V |
|  | NC Board of Nursing  NC Board of Pharmacy  NC Medical Board | Joint Statement on Pain Management in End-of-Life Care (1999) | HI; SPEC; PS | USA JPS I |
|  | Oncology Nursing Society | Oncology Nursing Society and Association of Oncology Social Work: Joint Position on End-of-Life Care (2003) | HI; EOL; PS | usa ons i |
|  |  | Nurse’s Responsibility to Patients Requesting Assistance in Hastening Death (2001, 2002, 2004, 2007) | HI; SPEC; PS | usa ons II |
|  | WV Board of Examiners for Registered Professional Nurses  WV Board of Medicine  WV Board of Osteopathy  WV Board of Pharmacy | Joint Policy Statement on Pain Management at the End of Life (2001) | HI; SPEC; PS | USA JPS II |

| **Representativeness**  **of the organization** | **Name**  **of the organization** | **Title of the document**  **and date of delivery** | **Type of the document** | **Document**  **code** |
| --- | --- | --- | --- | --- |
| National (UK) | National Council for Palliative Care | Palliative Care Defined | PCI; EOL; O | uk ncpc |
|  | St. Christopher’s Hospice | St. Christopher’s Mission Statement | PCI; EOL; PS | uk sc |
| National (AUSTRALIA) | Australian Medical Association | Position Statement on the Role of the Medical Practitioner in End of Life Care (2007) | HI; EOL; PS | AUSTRALIA AMA |
|  | Australian and New Zealand Society of Palliative Medicine | Joint Position Statement on Palliative Medicine in General Practice (2001) | PCI; EOL; PS | AUSTRALIA ANZSPM |
|  | College & Association of Registered Nurses of Alberta | Position Statement on Hospice Palliative Care (2005) | HI; EOL; PS | AUSTRALIA  CARNA |
|  | Palliative Care Australia | Strategic Plan 2003-2006 | PCI; EOL; O | australia  pca I |
|  |  | Position statement on Voluntary Euthanasia. Palliative care Practice and End of Life Decisions (2006) | PCI; SPEC; PS | australia  pca II |
|  |  | Carers and End of Life (*Interim Position Statement*, 2008) | PCI; SPEC; PS | AUSTRALIA  PCA III |
|  | Palliative Care Council of South Australia | Dealing with Dying | PCI; EOL; O | AUSTRALIA PCCSA |
