# Supplementary material for: What "best practice" could be in Palliative Care: an analysis of statements on practice and ethics expressed by the main Health Organizations
Source: BMC Palliat Care. 2010 Jan 7;9:1. doi: 10.1186/1472-684X-9-1 (PMC2823604; doi:10.1186/1472-684X-9-1)
Supplement: Additional file 2 — quotations. quotations from selected documents sorted by areas and subareas. [file 1472-684X-9-1-S2.DOC]

**Attachment 2: Quotations**

The tables below report the quotations from the documents, sorting quotations by “areas” and “sub-areas”.

| A – SYMPTOMS: **1 – Symptoms control (pain, diarrhea, vomiting, breathlessness, etc.)** | |
| --- | --- |
| **Quotation** | **Representativeness of the organization and**  **document code** |
| Control of pain, of other symptoms, and of psychological, social and spiritual problems is paramount.  One of the essential elements of a “good death” is freedom from the pain that dominates consciousness and may leave the patient physically and mentally incapable of reaching whatever goals he or she may want to achieve before death. There is thus no excuse for failure to use available methods to control pain adequately. | international  WHO I |
| Palliative care:   - provides relief from pain and other distressing symptoms; - (…). | international  WHO I, WHO V |
| In patients with advanced disease more that two thirds experience pain, and the management of pain and other symptoms becomes the main aim of treatment. (…) The aim of treatment is to relieve the pain to the patient’s satisfaction, so that he or she can function effectively and eventually die free of pain. | international  WHO II |
| (…) they [i.e. the dying children] should receive appropriate supportive and palliative care, including adequate pain control.  Severe pain in children with cancer is an emergency and should be dealt with expeditiously.  Palliative care for children dying of cancer should be part of a comprehensive approach that addresses their physical symptoms and their psychological, cultural and spiritual needs. It should be possible to provide such care in children’s own homes should they so wish.  Comprehensive care of children with cancer includes curative therapies, pain management, and symptom control plus compassionate support both for the children and for their families. | international  WHO IV |
| Palliative care is an approach that improves the quality of life of patients and their families facing the problem associated with life-threatening illness, through the prevention and relief of suffering by means of early identification and impeccable assessment and treatment of pain and other problems, physical, psychosocial and spiritual.  Major efforts should be made to achieve the highest coverage for pain relief and palliative care using low costs drugs (oral morphine) and other interventions.  Pain relief and palliative care must therefore be regarded as integral and essential elements of a national cancer control programme whatever the possibilities of cure. | international  WHO V |
| The duty of the physician is to heal, where possible, relieve suffering (…).  The clinical management of pain in terminal patients is of paramount importance in terms of alleviating suffering. | international  WMA I |
| Control of pain of other symptoms, and of social, psychological and spiritual problems is paramount. | international  EAPC I |
| Doctors have a professional and moral mandate to use every reasonable means available to free patients from the pain and other symptoms that cause them to suffer. | international  EAPC II |
| Make alleviation of pain and other physical symptoms a high priority. | international  ESMO |

| A – SYMPTOMS: **1 – Symptoms control (pain, diarrhea, vomiting, breathlessness, etc.)** | |
| --- | --- |
| Quotation | **Representativeness of the organization and**  **document code** |
| The alleviation of pain and suffering is a fundamental nursing responsibility and nurses are expertly trained in pain management, palliative care and in helping people in dealing with grief, death and dying. | international  ICN |
| The emphasis of care is on the “whole person” with symptom management as a well-recognised and integral part of hospice palliative care service delivery.  In providing pain and symptom management the nurse conducts a comprehensive assessment of pain and other symptoms using valid and developmentally appropriate tools.  The hospice palliative nurse:   - Identifies the multi dimensional factors that influence the person’s ‘total’ pain experience. - Recognizes that each person’s experience of pain is unique. - Advocates for appropriate pain management measures.   The hospice palliative care nurse:  - Administers the medications and or techniques that are appropriate to the severity and specific types of pain.  The hospice palliative care nurse:   - Demonstrates knowledge to assess and alleviate to the greatest extent possible the following common symptoms:   - Cardio-respiratory: dyspnea, cough, edema, hiccoughs, congestion.   - Gastrointestinal: candidiasis, mucositis, nausea, vomiting, constipation, obstipation, bowel obstruction, diarrhea, ascites, dehydratation, incontinence, jaundice   - General : anorexia, cachexia, fatigue, sleep disturbances, weakness, bleeding, odour, pruritus, bladder spasms, urinary retention, skin breakdown, seizures, mioclonus. | national  CANADA CHPCA I |
| Appropriate pain and symptom management is a key component of end-of-life care and addresses one of the common concerns expressed by dying individuals: a fear that they will experience pain and suffering.  Individuals and families living with life-threatening illness benefit from palliative care with its focus on pain and symptom management; social, psychological, emotional and spiritual support; and caregiver support. | national  CANADA CNA |
| Patients have the right to the best pain relief possible. | national  CANADA CPS |
| Ensure the alleviation of pain and management of other physical symptoms are a high priority. | National  USA AAFP I |
| Major concerns are pain and symptom management, information sharing and advance care planning, psychosocial and spiritual support, and coordination of care. | national  USA AAHPM I |
| Physical, emotional, psychosocial, and spiritual/existential domains of distress must be addressed.  The relief of pain and anxiety is an essential aspect of palliative care, and should be addressed throughout the course of illness. | national  USA AAP |
| Ensure alleviation of pain and management of other physical symptoms. | national  USA ACS |

| A – SYMPTOMS: **1 – Symptoms control (pain, diarrhea, vomiting, breathlessness, etc.)** | | |
| --- | --- | --- |
| **Quotation** | **Representativeness of the organization and**  **document code** | |
| Care for dying patients should focus on the relief of symptoms, not limited to pain, and should use both pharmacologic and non-pharmacologic means.  Within this document, palliative care refers to care directed toward the quality of life of patients who are dying including the relief of pain and other symptoms, attention to the psychological, emotional, social and spiritual needs of the patient, and the provision of support for the dying patient and the patient’s family.  Pain, anxiety, depression, dyspnea, constipation and other symptoms can all be significantly ameliorated, if not eliminated, in the vast majority of dying patients. | national  USA AGS | |
| Trustworthy assurances that physical and mental suffering will be carefully attended to and comfort measures intently secured. Physicians should be skilled in the detection and management of terminal symptoms, such as pain, fatigue, and depression, and able to obtain the assistance of specialty colleagues when needed. | national  USA AMA | |
| Nurses are essential to the assessment and management of pain and other distressing symptoms as they generally have the most frequent and continuous patient contact. In planning nursing care, patients have the right to appropriate assessment and management of pain and other distressing symptoms. Pain and symptom management must be respected and supported. (…) The nurse-patient relationship is a significant moral relationship, as such nurses must advocate on behalf of patients to ensure effective symptom control at the end of life.  When pain and other distressing symptoms are present, the patient should have appropriate and sufficient medication by appropriate routes to control symptoms, in whatever dosage and by whatever routes to control symptoms as perceived by the patient. | national  USA ANA | |
| This usually requires that the oncologist, primary care physician, or hospice physician be well versed in the techniques of palliative care, including the control of physical and psychological symptoms, e.g. control of nausea, anorexia, pain, diarrhea, constipation, fatigue, anxiety, and depression, and assume the role of team leader in coordinating the efforts of all other caregivers. | national  USA ASCO I | |
| A key component of quality cancer care is treatment of the individual with cancer including symptom management and psychosocial support. | national  USA ASCO II | |
| For imminently dying patients whose suffering is unrelenting and unendurable, palliative sedation may offer relief.   - affirm the value of end of life care that includes aggressive and comprehensive symptom management (…). - affirm the use of palliative sedation to manage refractory and unendurable symptoms in imminently dying patients as one method of aggressive and comprehensive symptom management (…). - all people, including vulnerable population such as infants, children, and the elderly, facing progressive, life-limiting illness have the right to optimal pain relief (…). - health care professionals must advocate for their patients to ensure adequate pain relief. | | national  USA HPNA I |
| Physicians, nurses and pharmacists should be knowledgeable regarding effective and compassionate pain relief, and patients and their families should be assured such relief will be provided. | national  USA JPS I | |
| Pain, other symptoms and side effects are managed based upon the best available evidence, which is skillfully and systematically applied. | national  USA NCP | |
| The test of palliative care lies in the agreement between the individual, physician(s), primary caregiver, and the hospice team that the expected outcome is relief from distressing symptoms, the easing of pain, and/or the enhancing the quality of life. | national  USA NHPCO I | |
| A – SYMPTOMS: **1 – Symptoms control (pain, diarrhea, vomiting, breathlessness, etc.)** | | |
| **Quotation** | **Representativeness of the organization and**  **document code** | |
| Hospice believes that death is an integral part of the life cycle and that intensive palliative care focuses on pain relief, comfort and enhanced quality of life as appropriate goals for the terminally ill.  Hospice addresses the needs and opportunities during the last phase of life by including the individual and family, trained volunteers, caregivers and clinical professionals in the caregiving team. This interdisciplinary approach to care focuses on the individual’s physical symptoms and the emotional and spiritual concerns of the patient and family. | national  USA NHPCO II | |
| Early identification and excellent assessment, reassessment, and treatment of pain and all other physical symptoms. | national  USA ONS I | |
| Provides relief from pain and other symptoms. | national  UK NCPC | |
| To offer relief from pain and other distressing symptoms. | national  UK SC | |
| Ensure that the patient is free from unnecessary suffering. | national  AUSTRALIA AMA | |
| The registered nurse (…) aims to improve quality of life through efforts to alleviate physical, emotional, psychological and spiritual suffering. | national  AUSTRALIA CARNA | |

| A – SYMPTOMS: **2 – Control of anxiety and other psychological symptoms (not dying with fear)** | |
| --- | --- |
| **Quotation** | **Representativeness of the organization and**  **document code** |
| Control of pain, of other symptoms, and of psychological, social and spiritual problems is paramount. | international  WHO I |
| The physiological basis of cancer pain includes a variety of mechanisms. The psychological aspects include anxiety, fear, depression and a sense of hopelessness. | international  WHO II |
| Palliative care for children dying of cancer should be part of a comprehensive approach that addresses their physical symptoms and their psychological, cultural and spiritual needs. It should be possible to provide such care in children’s own homes should they so wish. | international  WHO IV |
| Care of the dying extends beyond pain and symptom relief. It also supports the social, psychological and spiritual needs of the patients and their families.  Palliative care is an approach that improves the quality of life of patients and their families facing the problem associated with life-threatening illness, through the prevention and relief of suffering by means of early identification and impeccable assessment and treatment of pain and other problems, physical, psychosocial and spiritual. | international  WHO V |
| (…) more attention must be paid to developing palliative treatments and improving the ability of physicians to assess and address the medical and psychological components of symptoms in terminal illness. | international  WMA I |
| Psychiatric morbidity at the end of life is significant and causes substantial, potentially remediable suffering to dying patients and their families. Further, we believe that quality care for the psychiatric complications of terminal illness is and should be an integral component of excellent, comprehensive end-of-life care. | international  APM |
| Control of pain of other symptoms, and of social, psychological and spiritual problems is paramount. | international  EAPC I |
| A – SYMPTOMS: **2 – Control of anxiety and other psychological symptoms (not dying with fear)** | |
| **Quotation** | **Representativeness of the organization and**  **document code** |
| Assesses for and intervenes in psychosocial and spiritual issues related to pain.  To assess and alleviate to the greatest extent possible the following common symptoms:   - Cognitive: agitation, delirium, confusion, depression, hallucinations, paranoia. - Psychosocial and spiritual: anxiety, fear, anger, grief. | national  CANADA CHPCA I |
| Individuals and families living with life-threatening illness benefit from palliative care with its focus on pain and symptom management; social, psychological, emotional and spiritual support; and caregiver support. | national  CANADA CNA |
| Attention must focus on sources of physical, emotional, psychosocial, and spiritual distress. Sources of fear need to be addressed. | national  USA AAHPM IV |
| Palliative care is operationalized through effective management of pain and other distressing symptoms (…). | national  USA AAHPM V |
| Physical, emotional, psychosocial, and spiritual/existential domains of distress must be addressed.  The relief of pain and anxiety is an essential aspect of palliative care, and should be addressed throughout the course of illness. | national  USA AAP |
| Recognize, assess, and address psychological, social and spiritual problems. | national  USA ACS |
| Within this document, palliative care refers to care directed toward the quality of life of patients who are dying including the relief of pain and other symptoms, attention to the psychological, emotional, social and spiritual needs of the patient, and the provision of support for the dying patient and the patient’s family.  Pain, anxiety, depression, dyspnea, constipation and other symptoms can all be significantly ameliorated, if not eliminated, in the vast majority of dying patients. | national  USA AGS |
| Trustworthy assurances that physical and mental suffering will be carefully attended to and comfort measures intently secured. Physicians should be skilled in the detection and management of terminal symptoms, such as pain, fatigue, and depression, and able to obtain the assistance of specialty colleagues when needed. | national  USA AMA |
| This usually requires that the oncologist, primary care physician, or hospice physician be well versed in the techniques of palliative care, including the control of physical and psychological symptoms, e.g, control of nausea, anorexia, pain, diarrhea, constipation, fatigue, anxiety, and depression, and assume the role of team leader in coordinating the efforts of all other caregivers. (…) Psychological distress, including anxiety, depression, and suicide, have received little attention. Yet recent evidence suggests that these factors are important determinants of adverse outcomes among all patients, including terminally ill ones. | national  USA ASCO I |
| Psychological and psychiatric issues are assessed and managed based upon the best available evidence, which is skillfully and systematically applied. | national  USA NCP |
| A terminal illness can cause intense physical symptoms as well as fear of unrelieved symptoms. Individual may experience depression and hopelessness and may fear the loss of control over their person and environment. A potential “loss of self” requires that the dying are cared for by compassionate, sensitive, and knowledgeable professionals who will attempt to identify, understand, and meet individual needs. | national  USA ONS II |
| The registered nurse… aims to improve quality of life through efforts to alleviate physical, emotional, psychological and spiritual suffering. | national  AUSTRALIA CARNA |

| A – SYMPTOMS: **3 – Being assisted by a staff in order to make the process of dying more comfortable**  **(both physical and psychological)** | |
| --- | --- |
| **Quotation** | **Representativeness of the organization and**  **document code** |
| The social worker should be an integral member of the palliative care team, providing additional psychological for both patient and family and helping to identify and resolve practical social and financial problems.  Good palliative care depends on well trained health care staff who feel that they have sufficient psychological support to undertake emotionally demanding tasks. | international  WHO I |
| Evaluation is a vital first step in cancer management. It demands an understanding of not only the physical problem, but also the psychological social and spiritual component of the patient’s suffering. It is best achieved by a team approach. The responsibility for evaluation lies primarily with the physician, but certain components may be undertaken by other health care workers.  Evaluation and treatment of cancer pain are best achieved by a team approach. | international  WHO II |
| Palliative care uses a team approach to address the needs of patients and their families including bereavement counselling, if indicated. | international  WHO V |
| In the care of terminal patients, the primary responsibilities of the physician are to assist the patient in maintaining an optimal quality of life through controlling symptoms and addressing psychosocial needs, and to enable the patient to die with dignity and comfort. | international  WMA I |
| The hospice palliative care nurse believes:  Care is best provided through the collaborative practice of members of an interdisciplinary team to meet the physical, emotional, social and spiritual needs of the person and their family living with advanced illness. | national  CANADA CHPCA I |
| Nurses, as members of the interprofessional team, collaborate with the person, the person’s family and all those involved in providing care (such as physicians, other health-care professionals and volunteers) to support a holistic approach; incorporate the person’s priorities, values and choices in all aspects of care; and address any specific concerns that may arise. | National  CANADA CNA |
| Palliative care is comprehensive, specialized care provided by an interdisciplinary team to patient and families living with a life threatening or severe advanced illness expected to progress toward dying and where care is particularly focused on alleviating suffering and promoting quality of life. | national  USA AAHPM I |
| Assisting in this process is an interdisciplinary team of health care professionals, clergy and volunteers dedicated to providing the means for patient and family to remain free from intolerable suffering, whether physical, emotional, psychosocial or spiritual. | national  USA AAHPM IV |
| Pain and symptom control, psychological distress, spiritual issues and practical needs are addressed with patient and family throughout the continuum of care. | national  USA AAHPMA V |
| The provision of palliative care for children involves a partnership between the child, family, parents’ employer(s), teachers, school staff, and health care professionals, including nurses, chaplains, bereavement counsellors, social workers, primary care physicians, subspecialty physicians, and consultants. | national  USA AAP |
| Palliative care of dying patients is an interdisciplinary undertaking that attends to the needs of both patient and family. | national  USA AGS |

| A – SYMPTOMS: **3 – Being assisted by a staff in order to make the process of dying more comfortable**  **(both physical and psychological)** | |
| --- | --- |
| **Quotation** | **Representativeness of the organization and**  **document code** |
| An interdisciplinary team provides services to the patient and family, consistent with the care plan.  Comprehensive interdisciplinary assessment identifies the social needs of patients and their families, and care plan is developed in order to respond to these needs as effectively as possible. | national  USA NCP |
| Hospice provides support and care for persons in the last phases of an incurable disease so that they may live as fully and as comfortably as possible.  Physical, social, spiritual, and emotional care are provided by a clinically-directed interdisciplinary team consisting of patients and their families, professionals, and volunteers during the:   1. Last stages of an illness; 2. Dying process; and 3. Bereavement period. | national  USA NHPCO I |
| The interdisciplinary team should include the individual, family, registered nurses, physicians, other professionals, para-professionals and volunteers. | national  AUSTRALIA CARNA |

| B – RELATIONAL AND SOCIAL AREA: **1 – Respect of values and preferences; respect and acknowledgement of the cultural rules of dying (a death that happens within the current rituals of the cultural environment of reference).** | |
| --- | --- |
| **Quotation** | **Representativeness of the organization and**  **document code** |
| Patients have the right to expect that their spiritual experiences will be respected and listened to with attention (…) respect of people beliefs is imperative (…).  Supportive interventions in this area must be offered in ways that are non-sectarian, non dogmatic and in keeping with patient’s own views of the world. | international  WHO I |
| Palliative care for children dying of cancer should be part of a comprehensive approach that addresses their physical symptoms and their psychological, cultural and spiritual needs. | international  WHO IV |
| Be sensitive to and respectful of the patient’s and family’s wishes. | international  ESMO |
| Promote integration of the following into basic and post-basic nursing curriculum: skills in pain assessment and management, respect for cultural values, the right of dying patient to make informed decision, including the right to choose or to refuse treatment, and the right to a dignified death.  ICN supports national nurses associations in their endeavours to: support nurses dealing with complex palliative care issues related to death and dying, including compassionate and skilled care during the end stage of life, respecting the patient’s will and ethical standards and cultural norms in death and mourning (…). | international  ICN |
| Principles of autonomy, informed decision making, dignity, and respect for patients and their families will be paramount. | international  ISPN |
| The hospice palliative care nurses assesses the influence of cultural and spiritual values, beliefs, traditions, life way patterns of the person and family on their illness experience. | national  CANADA CHPCA I |
| B – RELATIONAL AND SOCIAL AREA: **1 – Respect of values and preferences; respect and acknowledgement of the cultural rules of dying (a death that happens within the current rituals of the cultural environment of reference).** | |
| **Quotation** | **Representativeness of the organization and**  **document code** |
| As patient are typically part of a family, when care is provided the patient and the family are treated as a unit. All aspect of care are provided in a manner that is sensitive to the patient’s and family personal, cultural and religious values, beliefs and practices their developmental state and preparedness to deal with the dying process. | national  CANADA CHPCA II |
| CNA recognizes a person’s right to make informed choices about his or her plan of care for end of life that reflect his or her personal, cultural and religious values. (…) CAN believes that nurses have an important role to play in encouraging all individuals, whether healthy or ill, to express their goals and wishes related to end-of-life care (…).  CAN believes that nurses, other health-care providers, and substitute decision-makers have an ethical and legal duty to respect any wishes that an individual previously expressed when capable.  Nurses should advocate for the communication of and respect for individuals’ goals and wishes across health-care settings as well as for the development of appropriate process and documentation that will facilitate this. | national  CANADA CNA |
| Be sensitive to and respectful of the patient’s and family’s wishes.  Use the most appropriate measures that are consistent with patient and surrogate choices. | national  USA AAFP I |
| The special needs of the pediatric and geriatric populations and patient’s cultural contexts are considered when formulating a comprehensive treatment plan. | national  USA AAHPM I |

| B – RELATIONAL AND SOCIAL AREA: **1 – Respect of values and preferences; respect and acknowledgement of the cultural rules of dying (a death that happens within the current rituals of the cultural environment of reference).** | |
| --- | --- |
| **Quotation** | **Representativeness of the organization and**  **document code** |
| Respect patient’s preferences for treatment, once the prognosis and anticipated trajectory with and without ANH have been explained. | national  USA AAHPM III |
| The central goal of hospice and palliative care is the relief of pain and suffering. In order to achieve this, a physical and social environment must be established conducive to comfort. This environment must include the patient’s personal and cultural values in the face of expected death.  The primary goal of end-of-life care must be alleviation of suffering while upholding respect for patients’ and families values. | national  USA AAHPM IV |
| Palliative care is operationalized through effective management of pain and other distressing symptoms while incorporating psychosocial and spiritual care according to patient/family needs, values, beliefs and culture(s). | national  USA AAHPM V |
| The provision of palliative care for children includes sensitivity to and respect for the child’s and family’s wishes. In consultation with the child’s parent or guardian, the plan of care incorporates respect for the terminally ill child’s preferences concerning testing, monitoring, and treatment. | national  USA AAP |
| The care of the dying patient, like all medical care, should be guided by the values and preferences of the individual patient. | national  USA AGS |
| The assessment and management of pain and other distressing symptoms must be based on an informed understanding of the individual patient’s values and goals and his/her emotional, physical and spiritual needs as well as on an understanding of the pathophysiology of the disease state and evidence based palliative care practice. | national  USA ANA |
| B – RELATIONAL AND SOCIAL AREA: **1 – Respect of values and preferences; respect and acknowledgement of the cultural rules of dying (a death that happens within the current rituals of the cultural environment of reference).** | |
| **Quotation** | **Representativeness of the organization and**  **document code** |
| Patients’ and families’ values and preferences regarding end-of-life care must be respected. | national  USA APS |
| Respect of patient’s values and preferences for end-of-life care. | national  USA ASPMN |
| Pain assessment and management should incorporate principles of cultural sensitivity as well as patient’s values and beliefs. | national  USA HPNA II |
| The care plan is based on the identified and expressed values, goals and needs of patient and family, and is developed with professional guidance and support for decision-making.  The physical environment in which care is provided should meet the preferences, needs and circumstances of the patient and family to the extent possible.  The palliative care program assesses and attempts to meet the culture-specific needs of the patient and family. | national  USA NCP |
| An individual’s needs must continue to be assessed and all treatment options explored and evaluated in the context of the individual’s values and symptoms.  The individual’s choices and decisions regarding care are paramount and must be followed at all times. | national  USA NHPCO I |
| The team works together to develop a plan of care and to provide services that will enhance the quality of life and provide support for the individual and family while respecting their wishes during the terminal phases of the illness and the bereavement period. | national  USA NHPCO II |
| To ascertain and honour the wishes, concerns, priorities and values of the patients and their families consistent with the law and the organization’s values as stated in its policies. | national  USA NHPCO III |
| Culturally competent practitioners who are able to respectfully inform patients and family about prognosis, interventions and outcomes within the context of differing value systems. | national  USA ONS I |
| Ensure that the patient’s goals and values for end of life care are respected. | national  AUSTRALIA AMA |
| Nursing care is provided through evidence-based practice, with sensitivity to personal, cultural religious values, beliefs and practice. | national  AUSTRALIA CARNA |
| To respect the worth and individuality of each person for whom we care. | national  UK SC |
| Understand and reflect the diversity of community attitudes to death and dying. | national  AUSTRALIA PCA I |
| Recognises and respects the fact that some people rationally and consistently request deliberate ending of life. | national  AUSTRALIA PCA II |

| B – RELATIONAL AND SOCIAL AREA: **2 – Emotional support provided to patients, their families and close friends** | |
| --- | --- |
| **Quotation** | **Representativeness of the organization and**  **document code** |
| Palliative care offers a support system to help the family to cope during the patient’s illness and in their own bereavement.  Family caregivers should be the patient’s main source of psychological support; they can be helped in this role by appropriate counselling. They should be made aware of the patient’s psychological needs and of common reactions to life-threatening illness. | international  WHO I |
| Comprehensive care of children with cancer includes curative therapies, pain management, and symptom control plus compassionate support both for the children and for their families. | international  WHO IV |

| B – RELATIONAL AND SOCIAL AREA: **2 – Emotional support provided to patients, their families and close friends** | |
| --- | --- |
| **Quotation** | **Representativeness of the organization and**  **document code** |
| Palliative care is the active total care of the child’s body, mind and spirit, and also involves giving support to the family. | international  WHO IV, WHO V |
| The person and family are identified as the unit of care, emphasizing their physical, emotional, psychosocial and spiritual needs.  The hospice palliative care nurse encourages and supports the expression of feelings, perceptions and fears, (..) respects the different emotional needs of the person and their family (…) assists the person and the family to manage the emotional response to the illness experience. | national  CANADA CHPCA I |
| Assisting in this process is an interdisciplinary team of health care professionals, clergy, and volunteers dedicated to providing the means for patient and family to remain free from intolerable suffering, whether physical, emotional, psychosocial or spiritual. | national  USA AAHPM IV |
| (…) bereavement support is available for the family. | national  USA AAHPM V |
| Pediatricians should support parental expression of the disappointment, anger, grief, and suffering associated with the child’s illness. | national  USA AAP |
| (…) assist the family in coping with the patient’s dying and death. Family members (with “family” defined by the individual patient) usually play a critical role in both providing care for the dying patients and in making decisions for dying patients who have lost decision-making capacity. Providing support for the patient’s family, including a period after the patient dies, is an important aspect of the care of dying patients. | national  USA AGS |
| Trustworthy assurances that care providers will assist the bereaved through early stages of mourning and adjustment. Patient and their loved ones should be able to trust that some support continues after bereavement. This may be by supportive gestures, such as a bereavement letter, and by appropriate attention to/referral for care of the increased physical and mental health needs that occur among the recently bereaved. | national  USA AMA |
| Currently inappropriate physician attitudes toward death, poor doctor-patient communication, inadequate understanding of palliative care techniques, and the insistence on active anticancer treatment beyond its usefulness pose significant barriers to improving end of life care.  These barriers can be overcome by providing better medical school and professional education in the many disciplines of palliative and end of life care, including pain management, recognition of when anticancer therapy will not help, effective communication skills, and psychosocial support. | national  USA ASCO II |
| A grief and bereavement program is available to patients and families, based on assessed need for services. | national  USA NCP |
| The integration of sensitive and appropriate psychosocial and spiritual care that promotes adequate coping for patients, significant others, and families across the illness continuum and extending through the bereavement period. | national  USA ONS I |
| Offer a support system to help the family cope during the patient’s illness and in their own bereavement. | national  UK NCPC |
| To offer a support system for family and friends during the dying person’s illness and in bereavement. | national  UK SC |
| Support not only the physical needs of the patient but also the psychological, emotional, religious, and spiritual needs of the patient and their family members and carers.  Provide counselling and other support to patients, their family members and carers throughout the patient’s condition, including support for family members and carers beyond the patient’s death. | national  AUSTRALIA AMA |

| B – RELATIONAL AND SOCIAL AREA: **2 – Emotional support provided to patients, their families and close friends** | |
| --- | --- |
| **Quotation** | **Representativeness of the organization and**  **document code** |
| “Palliative care is specialized health care of dying peoples which aims to maximize quality of life, and assist families and carers during and after death”. | national  AUSTRALIA ANZSPM |
| Hospice palliative care services include grief and bereavement support throughout the illness and following death.  The registered nurse (…) aims to improve quality of life through efforts to alleviate physical, emotional, psychological and spiritual suffering.  Hospice palliative care services include grief and bereavement support throughout the illness and following death. | national  AUSTRALIA CARNA |
| The scope of palliative care services includes grief and bereavement support for the patient and family and other carers during the life of the patient and after the patient’s death. | national  AUSTRALIA PCA II |
| Palliative care aims to (…) support patients, family and friends in approaching death and healing grief. | national  AUSTRALIA PCCSA |

| B – RELATIONAL AND SOCIAL AREA: **3 – Good communication between patient/families/close friends/caring staff.** | |
| --- | --- |
| **Quotation** | **Representativeness of the organization and**  **document code** |
| The psychological needs of the patient with advanced cancer (…) honest communication with family and care-givers, and confidence of receiving the best possible care. | international  WHO I |
| Il trattamento dei sintomi richiede organizzazione e comunicazione. Pazienti e famiglia dovrebbero essere informati sulla probabile causa del sintomo e su quali opzioni terapeutiche sino disponibili. Tali informazioni dovrebbero essere date chiaramente e in modo conciso con parole di facile comprensione. | international  WHO III |
| Health care professionals must establish trust through consistent honesty in their discussions with children and parents, particularly in answering all questions about death and dying. | international  WHO IV |
| Good communication is the key to psychological support. Imparting information must be undertaken with honesty and openness, in an atmosphere of sensitivity and compassion, with adequate emotional support (…). The level of information and pace at which it is given should be appropriate for an individual’s ability needs and culture. | international  WHO V |
| ISPN believes that psychiatric nurses have an obligation to provide the best possible mental health care at the end of life and for the remaining family after death. This includes (…) enhancing communication among caregivers, as well as patients and their family. | international  ISPN |
| Standard II: connecting. The hospice palliative care nurse establishes a therapeutic connection (relationship) with the person and their family through making, sustaining and closing the relationship. Making the connection. The hospice palliative care nurse:   - Communicates in a skilful and sensitive manner. - Listens actively as an integral part of communication. - Explains the role of nursing and other interdisciplinary team members. - Uses skills in verbal and non-verbal communication, listening and presence. - Demonstrates a comfort level in communicating with the person and family about issues related to death and dying. - Facilitates effective communication between person, family and care providers. | national  CANADA CHPCA I |
| Effective communication is fundamental to both the process of providing care and the function of a hospice palliative care organization. When combined with informed and skilled decision-making, it leads to better care delivery decisions, less conflict a more effective plan of care, greater patient/family/caregiver satisfaction with the therapeutic relationship, fewer caregiver errors, less stress and fewer burnout/retention problems. | national  CANADA CHPCA II |

| B – RELATIONAL AND SOCIAL AREA: **3 – Good communication between patient/families/close friends/caring staff.** | |
| --- | --- |
| **Quotation** | **Representativeness of the organization and**  **document code** |
| Developing and delivering a care plan for the end of life requires ongoing, open and honest communication; the timing and delivery of information should be tailored to the needs, developmental stage and readiness of the person and/or their family.  Nurses should advocate for the communication of and respect for individuals’ goals and wishes across health-care settings as well as for the development of appropriate process and documentation that will facilitate this. Nurses should also advocate for opportunities for revisiting, revising and documenting end-of-life care plans that can be clearly communicated across the continuum of health-care settings. | national  CANADA CNA |
| The AAFP believes that the highest-quality health care is an outgrowth of a partnership between the patient, the family, and the health professional or professional team. Within the context of this continuing relationship, family physicians must seek the underlying causes of suffering at the end of life, and then aggressively implement measures to correct them. | national  USA AAFP II |
| Optimal medical care of all patients, not just those who are dying, rests on frank and sensitive communication between patients and physicians. For dying patients, this ordinarily entails recognition and discussion of the facts surrounding prognosis and the likely course with a palliative plan of care. The conversation throughout must continue to elicit and respond to the patient’s needs. | national  USA AGS |
| The opportunity to discuss and plan for end-of-life care. This should include: the opportunity to discuss scenarios and treatment preferences with the physician and health care proxy, the chance for discussion with others, the chance to make a formal “living will” and proxy designation, and help with filing these documents in such a way that they are likely to be available and useful when needed. | national  USA AMA |
| Cancer care is based on truthful, sensitive, empathic communication with the patient, and in the case of paediatric patients, that care in both family centred as well as child focused. | national  USA ASCO I |
| Improve physician education. Currently inappropriate physician attitudes toward death, poor doctor-patient communication, inadequate understanding of palliative care techniques, and the insistence on active anticancer treatment beyond its usefulness pose significant barriers to improving end of life care. These barriers can be overcome by providing better medical school and professional education in the many disciplines of palliative and end of life care, including pain management, recognition of when anticancer therapy will not help, effective communication skills, and psychosocial support. | national  USA ASCO II |
| Interdisciplinary assessment of the patient to determine the refractory nature of his/her suffering and communication with the patient, family/ significant other/ surrogate decision maker, and other health care providers is critical to determine the appropriateness of palliative sedation and assure a valid informed consent process. | national  USA HPNA I |
| Communication and collaboration between members of the healthcare team, and the patient and family are essential in achieving adequate pain management in end-of-life care. | national  USA JPS I |
| Communication and collaboration between members of the healthcare team and with the patient and family are essential to achieve adequate pain control in end-of-life care. | national  USA JPS II |
| Good communication between the patient, their family members and carers, and the health care team may help alleviate fear, confusion, and guilt over the patient’s condition, assist decision-making, and reduce the potential for conflict over the patient’s care. | national  AUSTRALIA AMA |
| Every person has the right to participate in informed discussions about options and choices for care that can help optimize quality of life during the course of living with life-threatening illness.  Collaboration and effective communication among care providers is essential for quality care. | national  AUSTRALIA CARNA |
| | B – RELATIONAL AND SOCIAL AREA: **4 - Having close people nearby. Family acceptance of the patient’s condition.**  **Not feeling a burden for family and fiends.** | | | --- | --- | | **Quotation** | **Representativeness of the organization and**  **document code** | | The psychological needs of the patient with advanced cancer (…) the wish to feel needed and not to feel a burden.  All relatives and friends will need to discuss the events surrounding the death of the patient. This can be encouraged by attentive listening by a professional heath care worker or suitably trained volunteer. | international  WHO I | | Gli operatori sanitari dovrebbero assicurarsi che la famiglia sia preparata in anticipo ai problemi che potrebbero verificarsi e che sia in grado di affrontarli. | international  WHO III | | Parents need detailed guidance to lessen their worry and allow them to concentrate on enriching and sharing the last moments of their child’s life. | international  WHO IV | | A caring and supportive environment that acknowledges the inevitability of death helps family members to accept and deal with loss and grieving. | international  ICN | | Those working in palliative care share a common hope, that when the end of life come to us or to a loved one, it will be peaceful and free of pain, surrounded by those we love, feeling cared for and safe. | national  CANADA CHPCA I | | Care is only provided when the patient and/or family is prepared to accept it. | national  CANADA CHPCA II | | Patients may opt for treatment in the setting of their choice (usually at home) surrounded by supportive family members and friends. | national  USA AAHPM IV | | Sources of fear need to be addressed including patients’ perceptions of being an undue burden on family and caregivers. | national  USA AAHPM IV | | Maintaining control and not being a burden can also be relevant concerns. | national  USA AGS | | Trustworthy assurance that burden to family and others will be minimized. Patients should be able to expect sufficiently medical resources and community support, such as palliative care, hospice or home care, so that the burden of illness need not overwhelm caring relationship. | national  USA AMA | |  |

| **C – PREPARATION:**  **1 – Importance of preparation, awareness of diagnosis, awareness of dying.** | |
| --- | --- |
| **Quotation** | **Representativeness of the organization and**  **document code** |
| The psychological needs of the patient with advanced cancer:  (...) explanation of symptoms and the nature of the disease.  (...) the opportunity to discuss the process of dying. | international  WHO I |
| The hospice palliative care nurse initiates discussion with the person and family about issues related to meaning implication of diagnosis and prognosis dying and death. | national  CANADA CHPCA I |
| All aspect of care are provided in a manner that is sensitive to the patient’s and family personal, cultural and religious values, beliefs and practices their developmental state and preparedness to deal with the dying process. | national  CANADA CHPCA II |
| …..patients and families are thus helped not only to live comfortably during end-stage illness, but also helped to cope with and adequately prepare for inevitable death. | national  USA AAHPM IV |
| The effort to integrate palliative care into all health care for persons with debilitating and life-threatening illnesses should endeavour to ensure that: 4. Both patient and family are prepared for the dying process and for death, when it is anticipated. | national  USA AAHPM V |
| Regardless of the prognosis, respect for the child requires that he or she be given a developmentally appropriate description of the condition along with the expected burdens and benefits of available management options, while soliciting and listening to the child’s preferences.  As many children with chronic, life-shortening illnesses are now living into adolescence and young adulthood, the pediatrician needs to acknowledge the child’s own recognition of the likelihood of premature death, to help the child communicate his or her wishes, and to plan for the child’s death. | national  USA AAP |
| Attention to the personal goals of the dying person. Patients should be able to trust that their personal goals will have reasonable priority whether it be: to communicate with family and friends, to attend to spiritual needs, to take one last trip, to finish a major unfinished task in life, or to die at home or at another place of personal meaning. | national  USA AMA |
| Hospice exists in the hope and belief that through appropriate care, and the promotion of a caring community sensitive to their needs that individuals and their families may be free to attain a degree of satisfaction in preparation for death. | national  USA NHPCO I |
| People who have been told they are going to die of their illness need all kinds of information to help them prepare for death. | national  AUSTRALIA PCCSA |

| **C – PREPARATION:**  **2 – Choice of place of dying.** | |
| --- | --- |
| **Quotation** | **Representativeness of the organization and**  **document code** |
| The child and his or her family should decide where the final days are to be spent – in hospital, at home, or in a hospice.  Palliative care for children dying of cancer should be part of a comprehensive approach that addresses their physical symptoms, and their psychological, cultural, and spiritual needs. It should be possible to provide such care in children’s own homes should they so wish. | international  WHO IV |
| Care is provided in the setting that the person and family choose. (…) The hospice palliative care nurse believes:  - care should be provided, as much as possible, in the setting chosen by the person and family. | national  CANADA CHPCA I |
| Patients and families are assisted to select an appropriate setting of care. | national  CANADA CHPCA II |
| CAN believes that nurses have an important role to play in encouraging all individuals, whether healthy or ill, to express their goals and wishes related to end-of-life care (such as preferred location of death, …). | national  CANADA CNA |
| Patients may opt for treatment in the setting of their choice (usually at home) surrounded by supportive family members and friends. | national  USA AAHPM IV |
| The place where death occurs, whether in an intensive care unit, another area of the hospital, another institution, or at home, may depend on such factors as the wishes of the child and family, the physical layout and visitation policies of the alternative sites, the desire and ability of staff to remain involved, and the availability of other caregivers such as bereavement counsellors and clinicians with palliative care expertise. | national  USA AAP |
| Dying patients should be able to receive palliative care in the home, the hospital, and the nursing home, depending on the needs and preferences of the patient, without a disruption in the continuity of the patient’s care. | national  USA AGS |
| Attention to the personal goals of the dying person. Patients should be able to trust that their personal goals will have reasonable priority whether it be: to communicate with family and friends, to attend to spiritual needs, to take one last trip, to finish a major unfinished task in life, or to die at home or at another place of personal meaning. | national  USA AMA |
| The setting where care is to be provided and death occur is guided whenever possible by the preference of the person and family. | national  AUSTRALIA CARNA |

| **C – PREPARATION:**  **3 – Maintaining a sense of control (possibility of controlling relevant aspects of one’s own existence and/or deciding what and when to delegate to others). Maintaining a dimension of continuity of life right to the end.** | |
| --- | --- |
| **Quotation** | **Representativeness of the organization and**  **document code** |
| Palliative care offers a support system to help patients live as actively as possible until death; | international  WHO I, WHO III, WHO V |
| Every effort should be made to empower the patient and family by:   - involving them in decision-making with regard to treatment; - explaining treatments in such a way that they can give informed consent (or informed refusal); - facilitating a continuing sense of their being in control by providing appropriate advice and practical support. | international  WHO V |
| Palliative care provides expertise and standards of practice designed to (…) provide dying patients the tools to remain in control of their lives as well as their deaths.  ISPN supports the rights of patients to remain in control of their lives and their deaths. | international  ISPN |
| The dimension of “empowering” involves helping the person and family to uncover and build on their own strengths. The components of “empowering” include assessing the family assessing and supporting the person and family’s coping style, assisting with decision making and supporting choices made by the family, assisting the family to deal with ethical dilemmas, allowing the family to vent and defuse frustration, assisting in healing relationships, and giving information on all aspects of care.  The hospice palliative care nurse empowers persons and families to be active participants in their plan of care.  The hospice palliative care nurse provides care in a manner that is empowering for the person and family.  The hospice palliative care nurse assists the person and family to identify and build on their own strengths; establishes a plan of care in collaboration with the person and family; respects the person and family’s desire to seek therapies not offered in the conventional health care system.  The hospice palliative care nurse assists the person with advanced illness to address sensitive, personal issues related to sexuality and intimacy.  The hospice palliative care nurse assists the person and family to maintain a sense of control. | national  CANADA CHPCA I |

| **C – PREPARATION:**  **3 – Maintaining a sense of control (possibility of controlling relevant aspects of one’s own existence and/or deciding what and when to delegate to others). Maintaining a dimension of continuity of life right to the end.** | |
| --- | --- |
| **Quotation** | **Representativeness of the organization and**  **document code** |
| The patient may designate an alternate (proxy) decision maker, and specify when that person will act on his/her behalf (according to the laws in effect in the jurisdiction).  Each plan of care is customized, flexible and aims to support the patient’s and family’s desire for control, independence, intimacy, and their sense of dignity for as long as possible.  The patient can be as active in the delivery of care as s/he desires.  The setting of care in maintained so that it is safe, comforting, and provides ample opportunity for privacy and intimacy. Where possible, settings of care are homelike, with access to the outdoors. | national  CANADA CHPCA II |
| Maintaining control and not being a burden can also be relevant concerns.  The American Geriatrics Society (AGS) recognizes that most people near the end of life want to live as fully as they can. They want their health care providers to honour their wishes and goals and to help them maintain their dignity and independence while relieving symptoms and maximizing comfort. | national  USA AGS |
| The opportunity to discuss and plan for end-of-life care. This should include: the opportunity to discuss scenarios and treatment preferences with the physician and health care proxy, the chance for discussion with others, the chance to make a formal “living will” and proxy designation, and help with filing this documents in such a way that they are likely to be available and useful when needed. | national  USA AMA |
| Documented deficiencies in care at the end of life and the demographics of an aging population mandate a proactive approach to all facets of end-of-life care. Individuals with life-limiting illnesses and their caregivers deserve reliable and expert supportive care that improves quality of life and supports living as actively as possible until death. | national  USA ONS I |
| Offers a support system to help patients live as actively as possible until death. | national UK NCPC |
| The person decides who their *family* is. | national  AUSTRALIA CARNA |
| Palliative Care Australia believes (…) Enabling people’s preferences to receive quality care at the end of a life in the setting of their choice is dependent upon ongoing physical, emotional, practical and spiritual support from individual carers and their communities as well as health professional support. | national  AUSTRALIA PCA III |

| **D – EXISTENTIAL CONDITION:**  **1 – Being at peace with oneself and finding meaning.** | |
| --- | --- |
| **Quotation** | **Representativeness of the organization and**  **document code** |
| The spiritual aspect of human life may be viewed as an integrating component, holding together the physical, psychological and social components. It is often perceived as being concerned with meaning and purpose and, for those nearing the end of life, this is commonly associated with a need for forgiveness, reconciliation and affirmation of worth. | international  WHO I |
| The quality of care during the end stage of life greatly contributes to peaceful and dignified death and provides support to family members in dealing with their loss and grieving process. | international  ICN |
| The hospice palliative care nurse believes that a dignified and peaceful death is the right of all persons.  The dimension of “finding meaning “ involves helping the person and family make sense of their situation which is “strength-giving, and therefore empowering”. Davies and Oberle (1990) suggest “finding meaning” involves focussing on living and making the best of the situation by offering hope, as well as acknowledging and talking about death. A major component of this dimension is care of the person and family’s spiritual needs.  The hospice palliative care nurse assists the person to find meaning in their unique life and illness experience. | national  CANADA CHPCA I |
| Patients and families are thus helped not only to live comfortably during end-stage illness, but also helped to cope with and adequately prepare for inevitable death. Indeed, such care may facilitate significant growth and healing during the dying process. | national  USA AAHPM V |
| Spirituality and existential meaning. The dying process creates a myriad of questions and concerns about the meaning of life. People confront and resolve these questions and concerns in their own ways, frequently but not exclusively through religious and philosophical beliefs. Increasingly, those who care for the dying find that spiritual and existential issues are central to the quality of patients’ lives as they near death. | national  USA ASCO I |
| To help dying people with strong and unfamiliar emotions. To assist them to explore meaning, purpose and value in their lives. To offer the opportunity to reconcile and heal relationships and complete important personal tasks. | national  UK SC |
| Paring from those we love is the saddest thing most of us will ever face. But even a short time can be enough to do the things that have to be done – your Will if you do not already have one; the kind of funeral you would like the way your family affairs should be looked after; and letting people know what they mean to you. | national  AUSTRALIA  PCCSA I |

| **D – EXISTENTIAL CONDITION:**  **2 – Religious or spiritual practices and needs.** | |
| --- | --- |
| **Quotation** | **Representativeness**  **of the organization**  **and**  **document code** |
| Control of pain, of other symptoms, and of psychological, social and spiritual problems is paramount.  Because palliative care is concerned with the well-being of the whole person, it should acknowledge and respect the spiritual aspects of human life. Thus, all programmes of palliative care should respect and incorporate the basic values of spiritual and religious diversity that are enshrined in the United Nations’ Declaration on the elimination of all forms of intolerance and of discrimination based on religion and belief. (…) “Spiritual” refers to those aspects of human life relating to experiences that transcend sensory phenomena. This is not the same as “religious”, though for many people the spiritual dimension of their lives includes a religious components.  Patients who wish to participate in private or communal spiritual or religious activities must be enable to do so. At times, however, they will need privacy and access to spiritual advisers. | international  WHO I |
| Palliative care integrates the psychological and spiritual aspects of patient care; | international  WHO I, WHO V |
| Individual spiritual beliefs will often provide this inner strength, and spiritual care should be regarded as an important component of cancer care. Spiritual care may prove to be the most consistent and reliable source of comfort for children and their families.  Palliative care for children dying of cancer should be part of a comprehensive approach that addresses their physical symptoms, and their psychological, cultural, and spiritual needs. It should be possible to provide such care in children’s own homes should they so wish. | international  WHO IV |
| Palliative care is an approach that improves the quality of life of patients and their families facing the problem associated with life-threatening illness, through the prevention and relief of suffering by means of early identification and impeccable assessment and treatment of pain and other problems, physical, psychosocial and spiritual.  Care of the dying extends beyond pain and symptom relief. It also supports the social, psychological and spiritual needs of the patients and their families. | international  WHO V |
| Spiritual and religious issues, including both personal faith and relationship to a community of believers, are important for most people. Good end-of-life requires explicit attention to these matters. | international  APM |
| Control of pain of other symptoms, and of social, psychological and spiritual problems is paramount. | international  EAPC I |
| Dying persons and their families have individual cultural beliefs and values. Nurses must provide culturally sensitive and holistic care that respects spiritual and religious beliefs. | international  ICN |
| The person and family are identified as the unit of care, emphasizing their physical, emotional, psychosocial and spiritual needs. (…) The hospice palliative care nurse believes that facing death may be a personal or spiritual growth experience for each person. (…) The hospice palliative care nurse assists the person and families to have access to the appropriate resources to meet and address their spiritual needs. | national  CANADA CHPCA I |
| **D – EXISTENTIAL CONDITION:**  **2 – Religious or spiritual practices and needs.** | |
| **Quotation** | **Representativeness**  **of the organization**  **and**  **document code** |
| Individuals and families living with life-threatening illness benefit from palliative care with its focus on pain and symptom management; social, psychological, emotional and spiritual support; and caregiver support.  To provide quality end-of-life care, nurses must seek an understanding of the cultural, religious and other values and beliefs around death and dying that an individual and/or their family and/or their community may hold. To engage in conversations about death and dying with individuals in their care, nurses need to explore their own knowledge of, attitudes toward and beliefs around death and dying. | national  CANADA CNA |
| Major concerns are pain and symptom management, information sharing and advance care planning, psychosocial and spiritual support, and coordination of care. | national  USA AAHPM I |
| Assisting in this process is an interdisciplinary team of health care professionals, clergy, and volunteers dedicated to providing the means for patient and family to remain free from intolerable suffering, whether physical, emotional, psychosocial or spiritual. | national  USA AAHPM IV |
| Physical, emotional, psychosocial, and spiritual/existential domains of distress must be addressed.  The family must have the opportunity to carry out important family, religious, and/or cultural rituals and to hold the child before and after death. | national  USA AAP |
| Recognize, assess, and address psychological, social and spiritual problems. | national  USA ACS |
| Within this document, palliative care refers to care directed toward the quality of life of patients who are dying including the relief of pain and other symptoms, attention to the psychological, emotional, social and spiritual needs of the patient, and the provision of support for the dying patient and the patient’s family. | national  USA AGS |
| Attention to the personal goals of the dying person. Patients should be able to trust that their personal goals will have reasonable priority whether it be: to communicate with family and friends, to attend to spiritual needs, to take one last trip, to finish a major unfinished task in life, or to die at home or at another place of personal meaning. | national  USA AMA |
| The assessment and management of pain and other distressing symptoms must be based on an informed understanding of the individual patient’s values and goals and his/her emotional, physical and spiritual needs as well as on an understanding of the pathophysiology of the disease state and evidence based palliative care practice. | national  USA ANA |
| HPNA has developed this position statement to emphasize to healthcare systems and caregivers the importance of acknowledging and supporting the patient’s and family’s spiritual beliefs and expressions.  Patients and families are encouraged to display their own spiritual and religious symbols and should be able to practice their own spiritual and religious rituals in an accepting atmosphere. The use of religious symbols by staff or institutions should be sensitive to cultural and religious diversity.  Spiritual care necessitates the ability of the caregiver to reflect on and recognize the importance of one’s own spirituality and acceptance of the validity of others’ spiritual beliefs. One’s own values cannot be imposed on patients and families.  Effective spiritual care requires  (…) Recognizing and responding to another’s distress and helping them to discover meaning in their experiences of suffering, grief and loss.  It is the position of the HPNA Board of Directors to  Acknowledge that recognition of spirituality and spiritual distress is an essential component of palliative and hospice care.  (…) Recognize the right of the individuals to decline spiritual care. | national  USA HPNA III |

| **D – EXISTENTIAL CONDITION:**  **2 – Religious or spiritual practices and needs.** | |
| --- | --- |
| **Quotation** | **Representativeness**  **of the organization**  **and**  **document code** |
| Spiritual and existential dimensions are assessed and responded to based upon the best available evidence, which is skillfully and systematically applied. | national  USA NCP |
| Hospice addresses the needs and opportunities during the last phase of life by including the individual and family, trained volunteers, caregivers and clinical professionals in the caregiving team. This interdisciplinary approach to care focuses on the individual’s physical symptoms and the emotional and spiritual concerns of the patient and family. | national  USA NHPCO II |
| To remain sensitive to and be appreciative of the ethnic, cultural, religious, spiritual  and lifestyle diversity of patients and their families. | national  USA NHPCO III |
| The integration of sensitive and appropriate psychosocial and spiritual care that promotes adequate coping for patients, significant others, and families across the illness continuum and extending through the bereavement period. | national  USA ONS I |
| Integrates the psychological and spiritual aspects of patient care. | national  UK NCPC |
| Support not only the physical needs of the patient but also the psychological, emotional, religious, and spiritual needs of the patient and their family members and carers. | national  AUSTRALIA AMA |
| Nursing care is provided through evidence-based practice, with sensitivity to personal, cultural and religious values, beliefs and practices. | national  AUSTRALIA CARNA |

| **E – End of life decisions:**  **1 – Death as natural or normal/Not to hasten nor to postpone death** | |
| --- | --- |
| **Quotation** | **Representativeness**  **of the organization**  **and**  **document code** |
| Palliative care affirms life and regards dying as a normal process: neither hastens nor postpones death. | international  WHO I |
| Palliative care affirms life and regards dying as a normal process: intends neither to hasten or postpone death. | international  WHO V |
| Palliative care affirms life and regards dying as a normal process; it neither hastens nor postpones death. | international  EAPC I |
| The hospice and palliative care nurse believes:  - in the value of life and that death is a natural process.  The hospice palliative care nurse believes in the intrinsic worth of others, the value of live and that death is a natural process.  The hospice palliative care nurse acknowledges that death is natural life progression. | national  CANADA CHPCA I |
| The value of life, the natural process of death, and the fact that both provide opportunities for personal growth and self-actualization. | national  CANADA CHPCA II |
| Although increasing doses of pain medication may, in very limited instances, have a secondary and unintended effect of hastening death, this action is ethically justifiable if the dosage of pain medication is adjusted appropriately and the primary intent is to relieve pain. However, there is evidence that under-treatment of pain is a far more pressing concern. | national  CANADA CNA |
| Although a child’s life may be shortened by forgoing burdensome interventions or providing adequate sedation in the face of otherwise unrelieved symptoms, the goal of palliative care is to optimize the quality of the child’s experience rather than hasten death. | national  USA AAP |

| **E – End of life decisions:**  **1 – Death as natural or normal/Not to hasten nor to postpone death** | |
| --- | --- |
| **Quotation** | **Representativeness**  **of the organization**  **and**  **document code** |
| Palliative sedation is the monitored use of medications intended to induce varying degrees of unconsciousness, but not death, for relief of refractory and unendurable symptoms in imminently dying patients.  Thus, palliative sedation with its intent to relieve suffering in dying patients but not to deliberately hasten death is seen as distinct from euthanasia or assisted suicide where the intent is solely to end life. These statements reflect the rule of double effect. | national  USA HPNA I |
| Hospice affirms life and neither hastens nor postpones death. | national  USA NHPCO I |
| Aggressive symptom control to relieve unnecessary pain and suffering, which is consistent with a patient’s expressed wishes or values, is both legitimate and consistent with the ethical and legal practice of medicine, even if it may unintentionally hasten the patient’s death. | national  USA NHPCO V |
| Individuals with life-limiting illnesses and their caregivers deserve reliable and expert supportive care that improves quality of life and supports living as actively as possible until death. This model of care both affirms life and regards dying as a normal part of the life process. It intends neither to hasten nor postpone death. | national  USA ONS I |
| Affirms life and regard dying as a normal process. | national  UK NCPC |
| To affirm life; not to hasten death but to regard death as a normal process. | national  UK SC |
| Reaffirm that death is a natural part of life   - understand and reflect the diversity of community attitudes to death and dying - continue to refine the skills of the palliative care work force in supporting people to understand that death is a normal part of the life cycle - increase health professionals’ ability to work with death as a normal part of the life cycle. | national  AUSTRALIA PCA I |
| Believes that dying is a natural process and that declining or withdrawing futile treatment is acceptable. | national  AUSTRALIA PCA II |
| (…) death is recognized as part of the natural process of life. | national  AUSTRALIA CARNA |

| **E –End of life decisions:**  **2 - Death as an unwanted effect of sedation/Withdrawing or withholding treatments/**  **Euthanasia and assisted suicide.** | |
| --- | --- |
| **Quotation** | **Representativeness**  **of the organization**  **and**  **document code** |
| If shortening of life results from the use of adequate doses of an analgesic drug, this is not the same as intentionally terminating life by overdose. Any hastening of death that is linked to adequate pain control measures simply means that the patient could no longer tolerate the therapy necessary for a bearable and dignified life.  In many countries people have come to accept the notion that aggressive life-support, at intolerable personal cost is not the right course to take. From this has evolved the ethic of allowing terminally ill people to die peacefully, which may involve withholding of discontinuing such interventions as respiratory support, chemotherapy, surgery, and assisted nutrition.  It is ethically justifiable to withhold of discontinue life-support interventions that do not accord with a patient’s wishes when such interventions cannot reverse the course of disease but only prolong the process of dying. It is also ethically justifiable for doctors, after consultation with family members, guardians or proxies previously appointed by a patient, to take such decisions on behalf of unconscious or incompetent patients.  Drugs in the doses required to control pain and other symptoms should not be withheld solely because they may shorten a patient’s life. | international  WHO I |
| Providing adequate analgesia to a child is not the same as intentionally terminating his or her life, even in the unlikely event that adequate doses of pain-relieving drugs shorten life. | international  WHO IV |
| The patients right to autonomy in decision-making must be respected with regard to decisions in the terminal phase of life. This includes the right to refuse treatment and to request palliative measures to relieve suffering but which may have additional effect of accelerating the dying process | international  WMA I |
| Euthanasia, that is the act of deliberately ending the life of a patient, even at the patient’s own request of at the request of close relatives, is unethical. | international  WMA II |
| Physicians-assisted suicide, like euthanasia, is unethical and must be condemned by the medical profession. Where the assistance of the physician is intentionally and deliberately directed at enabling an individual to end his or her own life, the physician acts unethically.  However the right to decline medical treatment is a basic right of the patient and the physician does not act unethically even if respecting such a wish results in the death of the patient. | international  WMA III |
| In the course of illness, the time arrives when it is no longer possible to restore health, function or consciousness, and no longer possible to reverse the dying process. The most that even the aggressive use of sophisticated technology can achieve is to prolong the dying process. It is in these situations that we speak correctly of withholding of withdrawing interventions that are not stabilising a person’s life, but only prolonging a person’s dying. It is in these situations that we speak correctly of allowing a person to die. | international  EAPC II |
| The hospice palliative care nurse:  - supports informed choices that the person and family have made including withholding treatment, withdrawing life-sustaining therapies. | national  CANADA CHPCA I |
| The patient may request to have any therapy withdrawn at any time.  Requests to withhold or withdraw therapies, requests to initiate therapeutic interventions that appear to have no potential to benefit the patient and family, and requests to hasten death (i.e., euthanasia or assisted suicide) and the factor underlying those requests are discussed openly.  Caregivers respect the patient’s choices to have, withhold or withdraw therapeutic interventions. | national  CANADA CHPCA II |

| **E –End of life decisions:**  **2 - Death as an unwanted effect of sedation/Withdrawing or withholding treatments/**  **Euthanasia and assisted suicide.** | |
| --- | --- |
| **Quotation** | **Representativeness**  **of the organization**  **and**  **document code** |
| The plan of care should include current and future treatment options, as well as the right to refuse treatment. Refusal of treatment may involve a request that life-sustaining treatment, including artificial nutrition and hydration, be withdrawn or withheld.  Decisions around the withdrawing or withholding of treatments may involve the individual, the family, the health-care team and, if necessary, ethicists and ethics committees. Regardless of the outcome of decision-making around the withdrawing or withholding of treatments, nurses continue to provide quality end-of-life care and comfort measures. | national  CANADA CNA |
| As one of the many forms of palliative treatment, the use or sedating medications is intended to decrease a patient’s level of consciousness to mitigate the experience of suffering, but not to hasten the end of his or her life. Since this represents both an intention and an outcome that is beneficial, sedation in these cases is ethically justified. It is not analogous to euthanasia or physician-assisted suicide in which the primary intent is the death of the patient.  Although the withdrawal of artificial hydration and nutrition commonly accompanies sedation, the decision to provide, withdraw or withhold such treatments is separate from the decision whether or not to provide sedation.  The possibility that PS might hasten death as an unintended consequence should be assessed by the health care team in its consideration of PS, and then addressed directly in the process of obtaining informed consent. | national  USA AAHPM II |
| Acknowledge that ANH, like other medical interventions, can ethically be withheld or withdrawn, consistent with patient’s wishes and clinical situation. | national  USA AAHPM III |
| At times medication doses may be required that put a patient at risk for hastened death as an unintended effect. While such possibilities demand caution in treatment, one would still have acted ethically should death occur as a result of the treatment needed to relieve undesired symptoms. | national  USA AAHPM IV |
| Palliative care is operationalized through effective management of pain and other distressing symptoms (…).  Respect for autonomy includes the right to have interventions withdrawn or withheld upon request of the patient or designated surrogate, thus relieving the patient of interventions he pays to burdensome compared to expected benefits. The withholding or withdrawal of life-sustaining intervention is not considered euthanasia in current ethical and legal contexts. | national  USA AAHPM V |
| Each available diagnostic or therapeutic intervention needs to be considered within the context of the goals and expectations of the child and family. The decision to forgo certain treatments means that only those selected interventions are withheld or withdrawn.  The decision to forgo life-sustaining medical treatment does not necessarily imply an intent or choice to hasten the death of the child. Although a child’s life may be shortened by forgoing burdensome interventions or providing adequate sedation in the face of otherwise unrelieved symptoms, the goal of palliative care is to optimize the quality of the child’s experience rather than hasten death.  If a child or adolescent requests euthanasia, the health care team is to respond compassionately, with a renewed focus on determining and alleviating the sources of distress, including perceptions of abandonment, depression, loneliness, physical symptoms, and communication problems. | national  USA AAP |

| **E –End of life decisions:**  **2 - Death as an unwanted effect of sedation/Withdrawing or withholding treatments/**  **Euthanasia and assisted suicide.** | |
| --- | --- |
| **Quotation** | **Representativeness**  **of the organization**  **and**  **document code** |
| Respect the patient’s right to refuse treatment.  Recognize the physician’s responsibility to forego treatments that are futile. | national  USA ACS |
| Symptoms should be treated as vigorously as is appropriate to the patient’s situation and preferences to maximize comfort, even if the unintended effect of these efforts is, on rare occasions, the hastening of death. | national  USA AGS |
| Trustworthy assurance that preferences for withholding or withdrawing life-sustaining intervention will be honoured. Whether the intervention be less complex (such as antibiotics or artificial nutrition and hydration) or complex and more invasive (such as dialysis or mechanical respiration), and whether the situation involves imminent or more distant dying, patient’s preferences regarding withholding of withdrawing intervention should be honoured in accordance with the legally and ethically established rights of patients. | national  USA AMA |
| Regulations must specify that an intent to relieve pain, supported by documentation of the patient’s report of pain or behaviours that suggest pain (e.g., grimacing or moaning), can justify the use of high doses of analgesics or sedatives, even if these treatments also depress respiration or hasten death in some other way. Such treatment is based on ethicists and should not be considered an act of assisted suicide or euthanasia. | national  USA APS |
| ASCO believes that PAS (Patient Assisted Suicide) is a complex issue which this Task Force will neither condone nor condemn. | national  USA ASCO I |
| The use of palliative sedation requires that comfort be the priority goal of care. The use of cardiopulmonary resuscitation is generally viewed as inconsistent with this goal. However, decisions to withhold or withdraw other life sustaining therapies including artificial hydration and nutrition are separate from, but may be related to, the decision to use palliative sedation.  (…) palliative sedation with its intent to relieve suffering in dying patients but not to deliberately hasten death is seen as distinct from euthanasia or assisted suicide where the intent is solely to end life. | national  USA HPNA I |
| The goal of hospice is to provide people facing a life-limiting illness or injury with high quality, compassionate care that is expressly tailored to the patient's needs and wishes. Within the bounds of this goal, patients and their families have considerable latitude in the choices that they make regarding the patient’s care. One of these is the choice to withhold or withdraw artificial nutrition and hydration (ANH).  These decisions should ideally be made by a patient with decision-making capacity. Providers should be prepared to perform an assessment of decision-making capacity to ensure that the patient: 1) understands the medical situation and choices; 2) appreciates that the choice is relevant to him/her; 3) can weigh the risks and potential benefits of the decision; and 4) can express a choice that is consistent with his/her assessment of the relevant risks and potential benefits.  If the patient lacks decision-making capacity, choices may be made by a surrogate decision-maker with a knowledge of the patient’s preferences. Providers should obtain enough evidence of the patient’s wishes to be confident that the medical plan would have been consistent with his/her wishes.  If the patient lacks decision-making capacity and no surrogate decision-maker has any knowledge of the patient’s preferences, artificial nutrition and hydration may be withheld or withdrawn if doing so would be in the patient’s best interests.  In making recommendations, clinicians should be aware that hospice patients and their families are often heavily dependent on hospice providers for care. This relationship is particularly important in situations in which clinicians believe that withdrawal of ANH is appropriate and family members wish to continue. Under these circumstances, it is important that clinicians maintain their relationship with the patient and family, and continue to support the patient’s and family’s goals and values as much as possible while providing treatment that is medically appropriate.  • Decisions about artificial nutrition and hydration should be made in the same way as decisions about other treatments, by considering the patient’s preferences and the relevant risks and potential benefits.  • It is always acceptable to withhold or withdraw artificial nutrition and hydration when a competent patient refuses or when adequate evidence exists that an incompetent patient would have found the balance of risks and potential benefits to be unacceptable.  • It can be acceptable to withhold or withdraw artificial nutrition and hydration when doing so is in the patient’s best interests. | national  USA NHPCO IV |
| NHPCO Values Life: The philosophical constructs and evolving practices of hospice/palliative care are concerned foremost with the dignity of persons throughout the trajectory of life-limiting illness. When symptoms or circumstances become intolerable to a patient, effective therapies are now available to assure relief from almost all forms of distress during the terminal phase of an illness without purposefully hastening death as the means to that end. These modalities and the means to safely administer them must be within the expertise of and available from all hospice/palliative care providers as an alternative to PAS.  (…) the National Hospice and Palliative Care Organization supports improved knowledge of and access to hospice and palliative care for terminally ill people and their families, regardless of individuals’ views, decisions, or preferences regarding physician assisted suicide.  (…) the National Hospice and Palliative Care Organization does not support the legalization of physician assisted suicide. | national  USA NHPCO V |
| The AMA believes that while medical practitioners have an ethical obligation to preserve life, death should be allowed to occur with dignity and comfort when death is inevitable and when treatment that might prolong life will not offer a reasonable hope of benefit or will impose an unacceptable burden on the patient. | national  AUSTRALIA AMA |
| Believes that dying is a natural process and that declining or withdrawing futile treatment is acceptable. | national  AUSTRALIA PCA II |

| **E – End of life decision :**  **3 – Participation in the decision-making process.** | |
| --- | --- |
| **Quotation** | **Representativeness**  **of the organization**  **and**  **document code** |
| The psychological needs of the patient with advanced cancer: involvement in decision making, particularly as physical dependence on others increases. | international  WHO I |
| The patient’s right to autonomy in decision-making must be respected with regard to decisions in the terminal phase of life. | international  WMA I |
| A time comes in the course of a disease when it is wrong to continue to prolong life aggressively and when it is right to honour informed patients’ refusal of treatments that only prolong suffering.  If confusion, the brainwashing influence of other people and pathological depression can be excluded, then the will of the patient, and not health or survival alone, should govern decisions about initiating or discontinuing life prolonging treatment. Physicians are wrong to believe that they are obliged to insist on such treatment against the informed, free and stable refusal of a patient. | international  EAPC II |
| Promote integration of the following into basic and post basic nursing curriculum: (…) the right of dying patients to make informed decisions, including the right to choose or to refuse treatment, and the right to a dignified death. | international  ICN |
| Patients will be provided the opportunities of informed choice, input into decisions, and ability to change decisions as the situation changes. | international  ISPN |
| The hospice palliative care nurse believes that the person and family have the right to make informed decisions about all aspects of care, respecting the level of participation desired by the person and family. | national  CANADA CHPCA I |
| It is the patient’s right to: make informed decision and determine goals for care, establish priorities for present and future care from the available appropriate therapeutic options, change her/his mind at any time.  All patients are asked to designate a proxy decision-maker and specify under what circumstances that person should act. All patients are asked to provide advance directives to guide the proxy decision-maker should the patient become incapable of making decisions. When a patient lacks capacity to make decisions, approaches to decision making are guided by surrogate decision-making legislation and regulations in effect within the jurisdiction.  The patient may designate an alternate (proxy) decision maker, and specify when that person will act on his/her behalf (according to the laws in effect in the jurisdiction). | national  CANADA CHPCA II |
| CAN believes that nurses have an important role to play in encouraging all individuals, whether healthy or ill, to express their goals and wishes related to end-of-life care (such as preferred location of death, choices about organ donation, and whether or not they would like life-sustaining treatments such as cardiopulmonary resuscitation, artificial nutrition and hydration, dialysis and mechanical ventilation).  Nurses should encourage all individuals to document their wishes regarding care options, which may also include appointing someone to make decisions on an individual’s behalf should he or she become incapable. Capable individuals may change their mind about their goals and wishes at any time.  Persons who are deemed incapable, which may include young children, should be involved in decision-making to the extent that they are able. | national  CANADA CNA |
| **E – End of life decision :**  **3 – Participation in the decision-making process.** | |
| **Quotation** | **Representativeness**  **of the organization**  **and**  **document code** |
| The patient should give informed consent; if the patient is not capable of decision-making, the surrogate decision maker should give informed consent consistent with the goals of care and values previously stated by the patient. | national  USA AAHPM II |
| Establish open communication between patients/families and caregivers, to assure that their concerns are heard and that natural history of advanced illness is clarified. | national  USA AAHPM III |
| The ideal model of hospice care is one in which fully informed patients, having considered all feasible treatment options, determine that the potential burdens associated with curative interventions are outweighed by the anticipated benefits of care focused on symptom relief and quality of life. | national  USA AAHPM IV |
| Palliative care aims to guide and assist the patient and family in making decisions that enable them to work toward their goals during whatever time they have remaining. | national  USA AAHPM V |
| The informed decision of an adolescent or young adult patient nearing death to refuse further life-sustaining medical treatment ought to be respected (…). | national  USA AAP |
| In general, patients (or their surrogates) have the right to refuse treatment if they find its balance of risks, burdens, and potential benefits to be unacceptable.  If the patient lacks decision-making capacity, choices may be made by a surrogate decision-maker with a knowledge of the patient’s preferences. Providers should obtain enough evidence of the patient’s wishes to be confident that the medical plan would have been consistent with his/her wishes | national  USA NHPCO IV |
| The National Hospice and Palliative Care Organization supports the right of all persons to participate in all decisions regarding their care, treatment, and services | national  USA NHPCO V |
| The AMA supports the right of a competent patient to make fully informed health care decisions, including the right to refuse treatment. The AMA recognises that this may include life-sustaining treatment as well as palliative care. | national  AUSTRALIA AMA |
| Every person has the right to determine who has access to his/her personal information and who may participate in care decisions.  The registered nurse advocates for and involves the person and family in health care decision-making. | national  AUSTRALIA CARNA |
| The opportunity to discuss and plan for end-of-life care. This should include: the opportunity to discuss scenarios and treatment preferences with the physician and health care proxy, the chance for discussion with others, the chance to make a formal “living will” and proxy designation, and help with filing this documents in such a way that they are likely to be available and useful when needed. | national  USA AMA |
| Physicians should engage their patient in discussion about their concerns about how they might die, what comfort care will be provided, and give their patients assurance that they will not be abandoned. | national  USA ASCO I |
| It is particularly important that the physician frankly but sensitively discuss with the patient and the family their concerns and choices at the end of life. As part of discussion, the physician should make clear that, in some end of life care situations, there are inherent risks associated with effective pain relief. | national  USA JPS I |
| The right of the individual to make health care decisions based on personal values and quality of life considerations. | national  USA HPNA I |
| Patients have the right to participate actively in decision about their pain management. | national  USA HPNA II |

| **F - Quality of Life** | |
| --- | --- |
| **Quotation** | **Representativeness**  **of the organization**  **and**  **document code** |
| The goal of palliative care is achievement of the best possible quality of life for patient and their family.  Palliative care aims to maintain or improve the quality of life of patient with an incurable illness, and its impact may be evaluated by “measuring” quality of life. The starting point for such an evaluation is arbitrary. However, measures should assess reduction in suffering and changes in positive feelings and happiness. The components evaluated usually include physical symptoms, physical performance, psychological state and social interaction. Information provided by application of these measures in the context of palliative care is generally incomplete, because many important issues are not covered. Areas of omission include sexuality, the impact of illness on family structures, the “meaning” of illness to the patient, and the resolution of spiritual conflicts. | international  WHO I |
| The primary objectives of cancer treatment are cure, prolongation of life, and improvement of the quality of life. Improved quality of life is of paramount importance to patients with cancer, and pain relief and palliative care must therefore be regarded as integral and essential elements of an NCCP, whatever the possibilities of cure.  Palliative care is an approach that improves the quality of life of patients and their families facing the problems associated with life threatening illness, through the prevention and relief of suffering by means of early identification and impeccable assessment and treatment of pain and other problems, physical, psychosocial and spiritual.  Perhaps the most significant contribution of a national cancer control programme in this scenario is establishing a basis for pain relief and palliative care of individuals with advanced disease to ensure that they maintain a high as possible quality of life.  The quality of life dimensions of palliative care illustrated in Fig. 2 (physical, psychological and spiritual dimensions). Palliative care is concerned with not only all aspects of the patient’s need, but also the need of the family and of the health care provider. | international  WHO V |
| In the care of terminal patients, the primary responsibilities of the physician are to assist the patient in maintaining an optimal quality of life through controlling symptoms and addressing psychosocial needs, and to enable the patient to die with dignity and comfort. | international  WMA I |
| Palliative care sets out to preserve the best possible quality of life until death. | international  EAPC I |
| Palliative care provides expertise and standards of practice designed to improve the quality of patients’ lives (…). | international  ISPN |
| The focus on quality of life throughout the illness continuum, dying and bereavement.  The hospice palliative care nurse believes that each person and/or family defines their quality of life: promote, in the setting of advanced illness a quality of life and involves assisting persons to achieve their optimum state of health and well-being during illness and in the natural process of dying. Nurses have a unique and primary responsibility for advocating for the right of persons to maintain their quality of life for as long as possible and to experience a dignified and peaceful death.  The hospice palliative care nurse assist the person to achieve the best quality of life as defined by the person. | national  CANADA CHPCA I |

| **F - Quality of Life** | |
| --- | --- |
| **Quotation** | **Representativeness**  **of the organization**  **and**  **document code** |
| Care is guided by quality of life as defined by the individual. | national  CANADA CHPCA II |
| Palliative care is the provision of care aimed at relieving suffering and improving the quality of living and dying. | national  CANADA CNA |
| Palliative care is comprehensive, specialized care provided by an interdisciplinary team to patient and families living with a life threatening or severe advanced illness expected to progress toward dying and where care is particularly focused on alleviating suffering and promoting quality of life. | national  USA AAHPM I |
| In addition to alleviating pain and other physical symptoms, physicians must provide access to therapies that are likely to improve the child’s quality of life. Such therapies may include education, grief and family counselling, peer support, music therapy, child life intervention or spiritual support for both the patient and siblings, and appropriate respite care. | national  USA AAP |
| Provide access to therapies that may realistically be expected to improve the patient’s quality of life. | national  USA ACS |
| Within this document, palliative care refers to care directed toward the quality of life of patients who are dying including the relief of pain and other symptoms , attention to psychological, emotional, social and spiritual needs of the patient and the provision of support for the dying patient and the patient’s family. | national  USA AGS |
| When the restoration of health is no longer possible, the focus of nursing care is assuring a comfortable , dignified death and the highest possible quality of remaining life. | national  USA ANA |
| Cancer care optimizes quality of life throughout the course of an illness, through meticulous attention to the myriad physical, spiritual and psychosocial needs of the patient and family . | national  USA ASCO I, ASCO II |
| Hospice recognizes that the dying process is a part of the normal process of living and focuses on enhancing the quality of remaining life.  The National Hospice and Palliative Care Organization (NHPCO) defines palliative care as treatment that enhances comfort and improves the quality of an individual’s life during the last phase of life.  The test of palliative care lies in the agreement between the individual, physician(s), primary caregiver, and the hospice team that the expected outcome is relief from distressing symptoms, the easing of pain, and/or the enhancing the quality of life. | national  USA NHPCO I |
| Hospice affirms the concept of palliative care as an intensive program that enhances comfort and promotes the quality of life for individuals and their families.  Hospice believes that death is an integral part of the life cycle and that intensive palliative care focuses on pain relief, comfort and enhanced quality of life as appropriate goals for the terminally ill.  The team works together to develop a plan of care and to provide services that will enhance the quality of life and provide support for the individual and family while respecting their wishes during the terminal phases of the illness and the bereavement period. | national  USA NHPCO II |
| Documented deficiencies in care at the end of life and the demographics of an aging population mandate a proactive approach to all facets of end-of-life care. Individuals with life-limiting illnesses and their caregivers deserve reliable and expert supportive care that improves quality of life and supports living as actively as possible until death. | national  USA ONS I |
| The goal of palliative care is achievement of the best quality of life for patients and their families. | national  UK NCPC |
| Palliative care is specialized health care of dying peoples which aims to maximize quality of life, and assist families and carers during and after death. | national  AUSTRALIA ANZSPM |
| **F - Quality of Life** | |
| **Quotation** | **Representativeness**  **of the organization**  **and**  **document code** |
| Every person has the right to participate in informed discussions about options and choices for care that can help optimize quality of life during the course of living with life-threatening illness.  The registered nurse…aims to improve quality of life through efforts to alleviate physical, emotional, psychological and spiritual suffering.  Quality of life is defined by each person and their family (…). | national  AUSTRALIA CARNA |
| Palliative care is specialised health care of dying people which aims to maximise quality of life and assist families, carers and their communities during and after death. | national  AUSTRALIA PCA I |

| **G - Dignity** | |
| --- | --- |
| **Quotation** | **Representativeness**  **of the organization**  **and**  **document code** |
| The quality of care during the end stage of life greatly contributes to peaceful and dignified death and provides support to family members in dealing with their loss and grieving process. | international  ICN |
| The hospice palliative care nurse believes that a dignified and peaceful death is the right of all persons. | national  CANADA CHPCA I |
| Each plan of care is customized, flexible and aims to support the patient’s and family’s desire for control, independence, intimacy, and their sense of dignity for as long as possible. | national  CANADA CHPCA II |
| The American Geriatrics Society (AGS) recognizes that most people near the end of life want to live as fully as they can. They want their health care providers to honour their wishes and goals and to help them maintain their dignity and independence while relieving symptoms and maximizing comfort. | national  USA AGS |
| Trustworthy assurance that dignity will be a priority. Patients should be treated in a dignified and respected manner at all times. | national  USA AMA |
| The goal of hospice and palliative care is to facilitate safe and comfortable dying with focus on quality of life as each dying person defines it.  (…) the National Hospice and Palliative Care Organization reaffirms its commitment to the value of life and to the optimization of the quality of life for all people at the end of life. | national  USA NHPCO V |
| St. Christopher’s vision is of a world in which all dying people and those closed to them have access to appropriate care and support, when they need it, whenever they need it and whoever they are. Death must be approached in a way that respects the worth of each individual and that affirms and dignifies life. | national  UK SC |
| When the restoration of health is no longer possible, the focus of nursing care is assuring a comfortable, dignified death and the highest possible quality of remaining life. | national  USA ANA |
